# Experimental and computational insights into the CuFeMoO₄ modified CMC microbeads for effective removal of methylene blue from aqueous media

**DOI:** 10.1038/s41598-026-43208-1

**Published:** 2026-04-09

**Authors:** Mohamed A. Salem, Mohamed K. Awad, Rania K. Sleet, Marwa A. El-Ghobashy

**Affiliations:** Chemistry Department, Faculty of Science, Tanta University, Tanta, 31527 Egypt

**Keywords:** Microbeads, Catalysis, NaBH_4_, Methylene blue, Computational DFT, Td-DFT, NBO, Chemistry, Environmental sciences, Materials science

## Abstract

CuFeMoO₄/CMC microbeads were synthesized by crosslinking CuFeMoO₄ with carboxymethyl cellulose using AlCl₃ as a crosslinker. The microbeads were used as a catalyst in the reduction of methylene blue (MB) by sodium borohydride (NaBH_4_). The catalyst was characterized with FT-IR, XRD, BET, SEM, EDX, and TEM techniques. It has a surface area of 34.88 m^2^g^− 1^ and a pore diameter of 1.9212 nm. Within 45 min, the catalytic efficiency of the CuFeMoO_4_ composite increased from 25.8% to 99.2% after modification with carboxymethyl cellulose (CMC). The reduction rate constant followed the order CuFeMoO_4_/CMC (0.9609 ± 0.0233 min^− 1^) > CMC (0.2274 ± 0.00592 min^− 1^) > CuFeMoO_4_ (0.1365 ± 0.00445 min^− 1^). The catalytic reaction was carried under several reaction conditions, including catalyst dose, dye and NaBH_4_ concentrations, pH, NaCl concentration, temperature, and a radical scavenger. The results fit well with the pseudo-first-order kinetics. No significant change in the catalytic efficiency of the CuFeMoO₄/CMC up to the fifth cycle of reusability, demonstrating its high stability during the reaction. The basis function 6–31 G++ (d, p) in conjunction with the DFT method was applied for the computational investigation. Molecular modeling was carried out in both the gas phase and in water solvent to precisely predict the molecular structure, physicochemical properties, and spectroscopic spectra of MB, thereby validating the experimental results. Computational FT-IR, time-dependent-DFT (TD-DFT), natural bond orbital (NBO), and molecular electrostatic potential (MEP) were all calculated. The computational data and the experimental findings were in good agreement. Based on the regeneration data, CuFeMoO_4_/CMC proved a promising catalyst in the removal of MB from water.

## Introduction

The increased water pollution caused by rapid industrialization has become one of the main issues that threatens the lives of organisms and humans. The improper discharge of industrial wastewater containing various pollutants, such as antibiotics, pesticides, insecticides, heavy metals, and synthetic dyes, causes human disorders^1^. Among the dyes is Methylene blue (MB), which is known for its high solubility and stability. It is used in many industries such as painting, cosmetics, textiles, and pharmaceuticals^2^. The released dyes into the environment cannot only cause visible color even at low concentrations, but also reduce dissolved oxygen, which results in severe threats to living organisms, such as vomiting, confusion, shortness of breath, and headache^3^. Various techniques of water treatment, such as filtration, adsorption, coagulation, biodegradation, chemical, photochemical, electrochemical, and reverse osmosis, have been used for the removal of dyes^4^. The catalytic technique is considered an effective and popular way for the removal of pollutants from wastewater and hydrogen generation. This is due to its outstanding features, including environmental benignity, ease of operation, cost-effectiveness, no phase changing, high productivity, and high removal capacity. Therefore, the development of high-performance catalysts is the key to promoting their use in toxic wastewater control and hydrogen generation^5^. Metal borohydrides such as lithium borohydride, sodium aluminum hydride, and sodium borohydride have been paid attention because of their high hydrogen releasing capacities. Sodium borohydride (NaBH_4_) is a strong reducing agent that combines the best properties for storage and hydrogen generation, including solubility in water, rapid controllable hydrolysis, and overall stability^6^. During the hydrolysis of NaBH_4_, hydrogen can be generated as described in the reaction,                                                                                      

1 $$\mathrm{NaBH}_{4} + 2\mathrm{H}_{2}\mathrm{O} \rightarrow \mathrm{NaBO}_{2} + 4\mathrm{H}_{2} + \mathrm{heat} $$

Gvoić, et al. prepared the Fe_2_(MoO_4_)_3_ by a chemical wet procedure for oxidative degradation of Cyan flexo dye, where the highest degradation efficiency was achieved after 90 min at pH 2 and H_2_O_2_ concentration of 11 × 10^− 3^ molL^− 1^ ^7^. Dargahi, et al. Synthesized the Ce_2_(MoO_4_)_3_ by microemulsion method in the presence of two different surfactants, cetyltrimethylammonium bromide (CTAB) and Triton X-100, for Crystal violet removal^8^. During 5 h, Ce_2_(MoO_4_)_3_ removed about 89% of Crystal violet under visible light irradiation. Nobakht et al. removed the phenol red dye from wastewater by the activated carbon/Fe_2_(MoO_4_)_3_ composite as an adsorbent^9^. The highest performance of AC/Fe_2_(MoO_4_)_3_ was observed at pH 3, adsorbent dose 0.05 g, dye concentration 10 mgL^− 1^, contact time 60 min, and 25 °C. Wang, et al. synthesized the Ag_2_MoO_4_ (AMO) assembled onto BiOBr nanosheets (BOB) for methylene blue degradation^10^. The mechanism of AMO/BOB composite depended on its photocatalytic activity, where h^+^, OH^•^, and O^•2−^ species were verified as the dominant free radicals inducing the dye degradation. Lin, et al. employed FeMoO_4_ to activate persulfate (S_2_O_8_^2−^) for the degradation of the azo dye Orange G (OG)^11^. More than 95% of this dye was eliminated after 40 min. Syah, et al. prepared CuMoO_4_/ZnO nanocomposite as a photocatalyst to break down water contaminants, including methylene blue and Rhodamine B^12^. The produced CuMoO_4_/ZnO nanocomposite showed an outstanding photocatalytic performance and destroyed the methylene blue and Rhodamine B at 92% and 84%, respectively, after 70 min in the presence of UV irradiation. Hassani, et al. synthesized the α-CuMoO_4_^13^ as a catalyst for the oxidation of methylene blue with hydrogen peroxide and also for the reduction of para- meta- and ortho-nitrophenols.

Carboxymethyl cellulose (CMC) is an anionic macromolecular polysaccharide with numerous carboxylic and hydroxyl groups. It has been applied in medical, pharmaceutical, and water treatment^14^. The anionic groups in the CMC not only improve its solubility but also enhance its efficiency for the removal of cationic contaminants through the ionic interaction process. Several modifications, such as grafting, crosslinking, and composite formation, have been made for improving the mechanical stability and adsorption properties of CMC^15^. The CMC hydrogels have been considered a preferable form for CMC-based adsorbents owing to their chemical stability, easy separation, and sizable area available for adsorption^16^. Mostafa, et al. synthesized agar-carboxymethyl cellulose–silver (AG-CMC-Ag) nanocomposite by modification of cellulose to CMC with silver and agar^17^. This nanocomposite was applied for the removal of methylene blue, which exhibited a maximum removal capacity of 66.6 mgg^− 1^. Eltaweil, et al. removed the methylene blue by carboxymethyl cellulose/carboxylated graphene oxide (CMC/GOCOOH)^18^. This nanocomposite gave a maximum adsorption capacity of 183.1 mgg^− 1^. It was stable and active after nine successive reusable cycles towards MB removal. Tanzifi, et al. increased the adsorption capacity of polypyrrole by using carboxymethyl cellulose^19^. The PPy/CMC nanocomposite was used for the removal of Reactive blue 160 and Reactive red 56 dyes, which exhibited maximum adsorption capacities of 104.9 and 120.1 mgg^− 1^ for RR56 and RB160, respectively. Radoor, et al. synthesized the PVA/CMC/halloysite nanocomposite for the degradation of methylene blue, with a maximum adsorption capacity of 40.6 mgg^− 1^ ^20^. Zhao et al. used polyvinyl alcohol/carboxymethyl cellulose/copper sulfide (PAM/CMC/CuS) hydrogel for the degradation of Rhodamine B and Neutral red dyes^21^. The hydrogel showed a maximum adsorption capacity of 208.3 mgg^− 1^.

Computational chemistry has developed into a powerful tool in chemistry, offering comprehensive data for atomic-level research of reaction mechanisms. When compared to alternative methods, the density functional theory (DFT) is a unique, cost-effective, and exceptional technique that produces precise results^22–26^. The DFT reproduced with exceptional precision the following parameters: geometry, dipole moment, vibration frequency, chemical shifts, molecular electrostatic potential (MEP), and frontier orbitals^27,28^. Amrhar, et al.^29^ demonstrated the significant ability of the MB molecule to adsorb onto the surface of NMC. Al-Wasidi, et al.^30^ utilized zeolitic imidazolate-8 metal-organic framework (ZIF-8) for the removal of MB. Guediri, et al.^31^ prepared orange peels treated with phosphoric acid (OP-H_3_PO_4_) to eliminate MB. The B3LYP hybrid functional at 6-31G(d, p) basis set was employed in the DFT to geometrically optimize the molecular structure of MB.

In this paper, the novel CuFeMoO₄/CMC nanocomposite was first synthesized as an efficient catalyst for the reduction of MB with NaBH₄ in aqueous solution. The synthesis of CuFeMoO₄/CMC microbeads offers a clear advantage in terms of life-cycle, scalability, and cost-effectiveness. The synthesis method relies on abundant and inexpensive elements (Cu, Fe, Mo) and a biodegradable polymer (CMC). Compared to the noble metals such as Ag, Au, or complex perovskite systems, this facile and economically viable route is amenable to scale-up, and the expected high catalytic efficiency supports its potential for long-term application^32,33^. The novelty of the work, therefore, lies in the first-time preparation of such a low-cost and highly effective nanocomposite for catalytic water purification, establishing a promising foundation for future materials designed to eliminate hazardous pollutants. The DFT calculations correlate the theoretical insights with the experimental findings.

## Materials and methods

### Materials and equipment

Methylene blue dye (C_16_H_18_ClN_3_S) was purchased from Sigma-Aldrich and used as received. Copper chloride (CuCl_2_·2H_2_O, 99%), ferric nitrate (Fe(NO_3_)_3_.9H_2_O, 99.5%), aluminum chloride (AlCl_3_, 99%), ammonium molybdate ((NH_4_)_6_Mo_7_O_24_.4H_2_O, 99%), and oxalic acid (C_2_O_4_H_2_.2H_2_O, 99.5%) were obtained from Merck (Darmstadt, Germany). Carboxymethyl cellulose (CMC) was purchased from Sigma-Aldrich. All these chemicals were used as received. Distilled water was used during the experimental work.

The catalyst was analyzed by various instruments, where its functional groups were characterized by a Fourier Transform Infrared spectrophotometer (JASCO FT-IR-4100, Japan) within the range 4000–400 cm^− 1^. The crystal structure was analyzed by powder X-ray diffraction (XRD). The XRD study was carried out using a Bruker D8 (Germany). A copper Ka radiation target of wavelength 1.54 Å was employed. The specific surface area and the pore size were measured using the Brunauer-Emmett-Teller (BET) and Barrett-Joyner-Halenda (BJH) methods (St 3 on NOVA touch 4LX [s/n:17016062702]) after the sample was degassed at room temperature for 3 h. The surface morphology was determined by a Scanning Electron Microscope (SEM) (NRC, QUANTA FEG250, Japan) and the Energy Dispersive X-ray (EDX) analysis was performed with IT100LA that attached to the SEM and operated at an accelerating voltage of 20.00 keV. The TEM micrographs were taken by a Transmission Electron Microscope (JEM-2100 and JEOL, Japan). The pH measurements were carried out by an AD8000 pH meter. An Ultraviolet/Visible (UV/Vis) double beam spectrophotometer (Shimadzu 2100-S, Japan) was used for the spectral measurements of the dye.

### Preparation of copper iron molybdate catalyst (CuFeMoO_4_)

CuFeMoO_4_ was prepared by complex thermal decomposition, where in the solid state, ferric nitrate, copper chloride, ammonium molybdate, and oxalic acid were mixed homogeneously in a mortar with a molar ratio of 2: 1: 0.43:10, respectively. Subsequently, the homogeneous mixture was heated for 1 h at 160 °C. Its color changed from orange to black during heating, indicating the formation of copper iron molybdate complex. The complex was left to cool to room temperature, then calcined in a muffle furnace at 500 °C for 2 h. After multiple washings, the composite was left to dry for 24 h at 60 °C.

### Preparation of CuFeMoO_4_/CMC microbeads

The CMC (0.75 g) was left to dissolve in 25 mL of water for 1 h at 60 °C. The CuFeMoO_4_ catalyst (0.25 g) was added to 10 ml of water and sonicated for 15 min. The CuFeMoO_4_ was then added to the CMC suspension and stirred for 1 h with heating. To form the microbeads, an AlCl_3_ crosslinking solution (3%) was prepared, and the uniform mixture of CuFeMoO_4_/CMC was transferred into the AlCl_3_ solution via a 3 cm³ syringe. Magnetic stirring was used to add the mixture dropwise for about 30 to 40 min. The product was filtered, washed several times with water, and dried overnight at 30 °C.

### Catalytic reduction of methylene blue

To study the catalytic activity of CuFeMoO_4_/CMC microbeads, the MB and NaBH_4_ (reducing agent) solutions were mixed with stirring for 2 h, whereby no decolorization of the dye was observed. The microbeads were then added to the mixture solution, and the time was noted. The catalytic reduction of MB was followed by measuring the decrease in absorbance with time. The concentration of the NaBH_4_ solution was kept significantly greater than that of the dye. In a typical reaction, the mixture consisted of H_2_O (48.4 mL), MB (1.1 mL of 1 × 10^− 3^ mol L^− 1^ solution), NaBH_4_ (0.5 mL of 0.1 mol L^− 1^ solution), and a nanocomposite dose of 0.02 g. The kinetics measurements began immediately once the catalyst was added to the reaction mixture. The decrease in absorbance of the unreacted dye was recorded in a 1-cm quartz cell. The measurements were continued till no further decrease in absorbance was observed.

## Results and discussion

### Characterization of the catalyst

#### Fourier transform infrared (FT-IR)

**Fig. 1:**
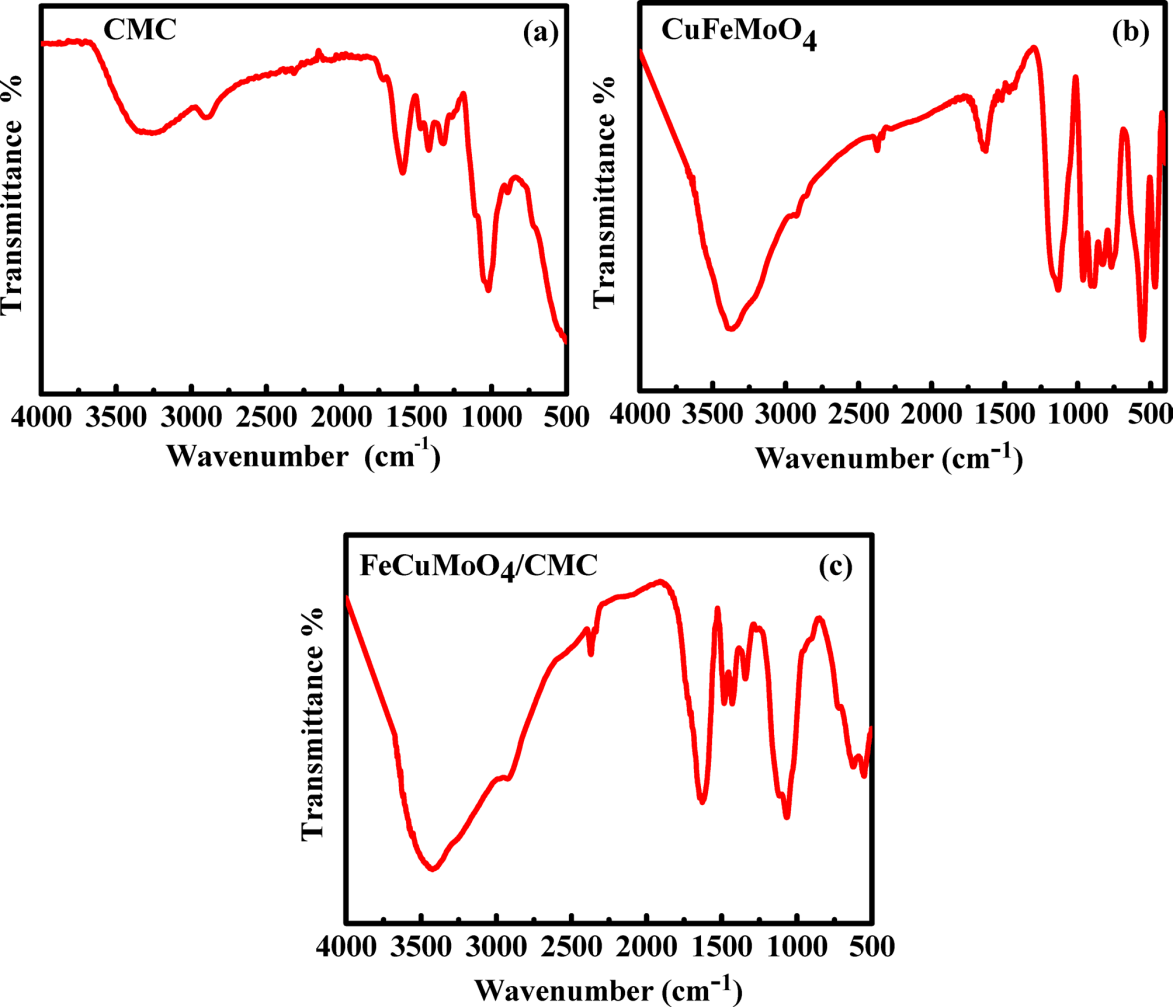
FT-IR spectra of (**a**) carboxymethyl cellulose, (**b**) CuFeMoO_4_ composite, (**c**) CuFeMoO_4_/CMC microbeads.

Figure 1 represents the FT-IR spectra of CuFeMoO_4_, CMC, and CuFeMoO_4_/CMC. For CuFeMoO_4_, the intense peak at 530–560 cm^− 1^ is related to the Fe-O and Cu-O. The peak at 914–955 cm^− 1^ is characteristic of the Mo-O-Mo stretching band. The peak at 1400 cm^− 1^ is assigned to the C-O stretching band and the peak at 1600–1709 cm^− 1^ is related to the C = O stretching band of the oxalate group. The broad band at 3400 cm^− 1^ is attributed to the OH stretching band^13,34^. For the CMC, the broad band at 3250 cm^− 1^ corresponds to the OH stretching band while the peak at 2900 cm^− 1^ is assigned to the C-H stretching band. The peak at 1422–1624 cm^− 1^ is attributed to the COO group. The peak at 1333 cm^− 1^ is characteristic of C-O-C. The peak at 1016 cm^− 1^ is related to the C-O stretching band in CMC^35^. These results proved the successful formation of the CuFeMoO_4_/CMC nanocomposite.

### X-ray diffraction (XRD)

**Fig. 2:**
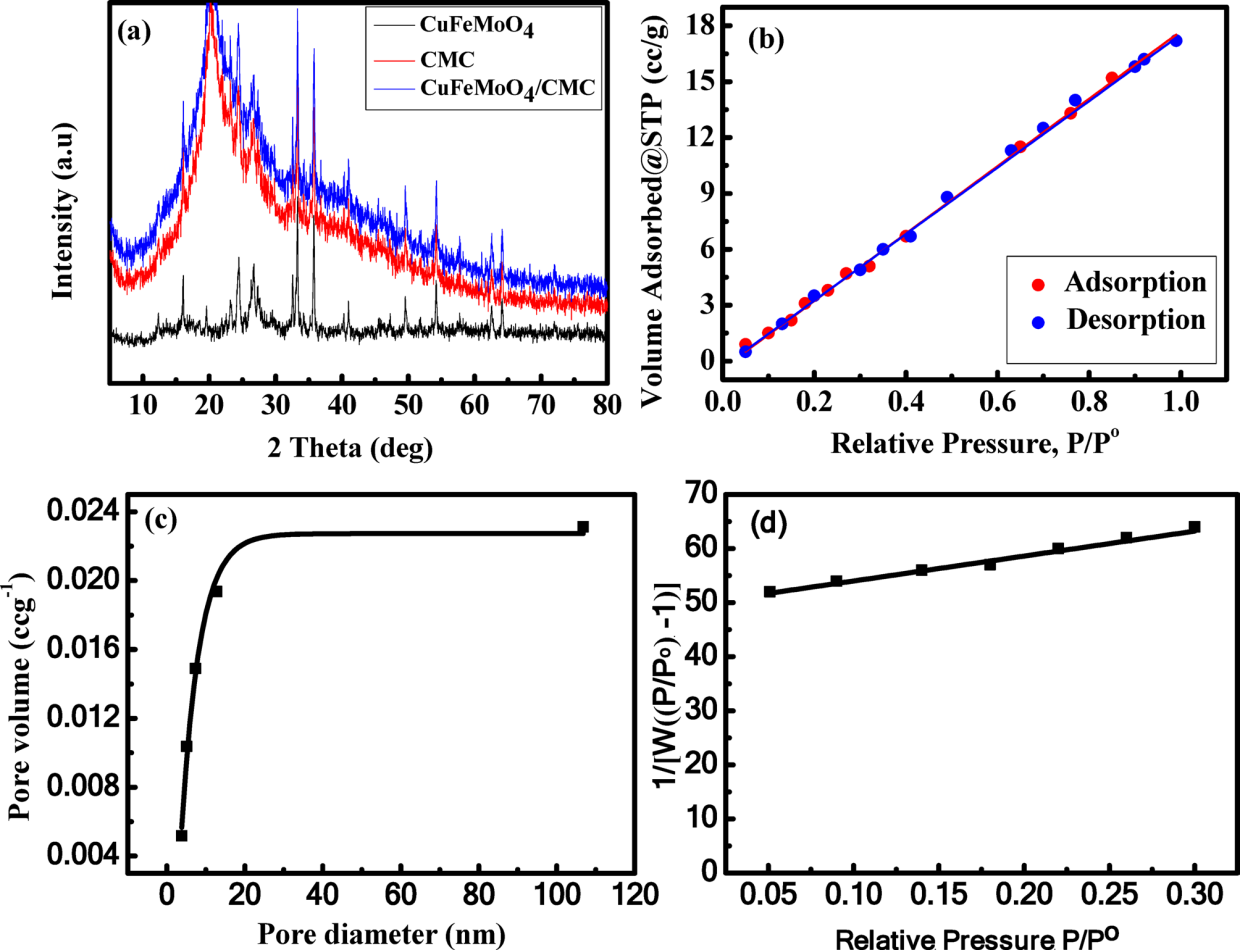
For CuFeMoO_4_/CMC microbeads: (**a**) XRD, (**b**) N_2_ adsorption-desorption isotherm at 77.3 K, (**c**) pore diameter distribution, (**d**) multi-point plot of CuFeMoO_4_/CMC microbeads.

The XRD analysis demonstrate the effective production of CuFeMoO_4_/CMC microbeads. Figure 2(a) represents the XRD patterns of CuFeMoO_4_, CMC, and CuFeMoO_4_/CMC microbeads. The FeCuMoO_4_ pattern shows that the sample primarily consists of a β-FeMoO_4_ phase, with a trace quantity of α-FeMoO_4_ and α-CuMoO_4_. The (110), (220), (112), (202), and (330) planes of β-FeMoO_4_ can be associated with the peaks at 12.9°, 26.2°, 31.6°, 27.33°, and 39.7°, respectively. The other peaks at 21.3°, 28.2° and 40.9° arise from α-FeMoO_4_ while the peaks of α-CuMoO_4_ appear at 2θ = 35.6° and 38.9°. A good degree of crystallinity is indicated by the strength of the XRD peaks, which is crucial for the sample’s catalytic activity^13,36,37^. The XRD of CMC shows a single-phase structure at 2θ = 20°, indicating an amorphous structure for CMC^38^. The observation of several crystal diffraction peaks during the synthesis of CuFeMoO_4_/CMC from the CuFeMoO_4_ and CMC suggests that the reaction between the two materials altered their initial crystal structures, confirming the formation of CuFeMoO_4_/CMC gel microspheres^35,39^.

### Brunauer-emmett-teller (BET)

The BET is the most important method for determining the surface properties of the composite. The N_2_ adsorption-desorption isotherms at 77 K for CuFeMoO_4_/CMC microbeads are presented in Fig. 2 (b). The inset showing their corresponding Barrett–Joyne–Halenda (BJH) pore-size distribution indicates that the average pore diameter of CuFeMoO_4_/CMC is 1.9212 nm, Fig. 2(c). The isotherm can be classified as a type IV isotherm. According to the investigation results, the CuFeMoO_4_/CMC microspheres gave rise to a specific surface area of 34.88 ± 0.23508 m^2^g^− 1^ and a pore volume of 0.266 ± 0.002 cm^3^g^− 1^, Fig. 2(d).

### Scanning electron microscopy (SEM)

**Fig. 3:**
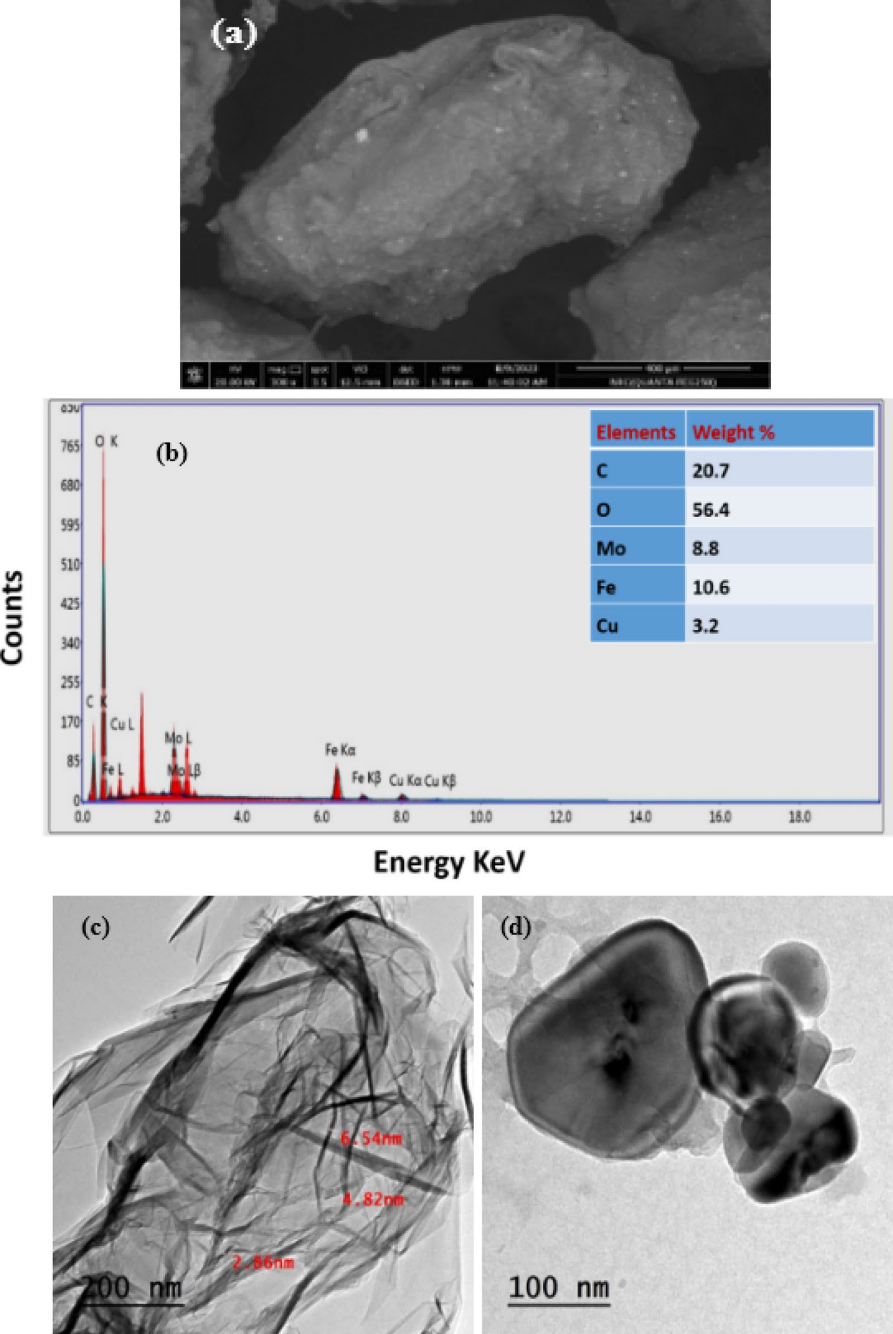
(**a**) SEM micrographs, (**b**) EDX analysis, and (**c**&**d**) TEM micrographs of CuFeMoO_4_/CMC microbeads before MB reduction.

Figure 3(a) depicts the surface morphology of CuFeMoO_4_/CMC microbeads. The microbeads display a crusty surface with some wrinkles. This outcome was consistent with interfacial interactions between the CMC chains and CuFeMoO_4_ nanoparticles, which may serve as intermolecular crosslinkers. The cross-section revealed that the rough-surfaced beads were comparatively spherical in shape and have a compact, smooth, and homogeneous cross-section. Additionally, all of the tested beads’ perfect spherical form was obviously altered by the water loss, and some wrinkles and fractures were seen as a result of the hydrogel polymer network partially collapsing during the drying process^18,40^.

### EDX analysis

Figure 3(b) illustrates the presence of Fe, Cu, Mo, and O for CuFeMoO_4_, C and O for the CMC, indicating the successful formation of the CuFeMoO_4_/CMC microbeads^41^.

### Transmission electron microscopy (TEM)

The TEM micrographs depicted in Fig. 3(c, d) reveal that the CuFeMoO_4_ nanoparticles are well dispersed in the CMC matrix to form a nanorod-like structure with an average diameter of 5 nm. The microbeads are almost spherical and have a smooth surface. The crystallographic and morphological discrepancies of the prepared microbeads were determined from the TEM micrographs. Additionally, the TEM results demonstrate that when the inner cores of CuFeMoO_4_ nanoparticles were wrapped in the CMC to harden in AlCl_3_, the nanoparticles were uniformly sealed with the CMC. The CuFeMoO_4_ attracted each other to form a more compact and stable sphere^42,43^.

### Effect of different parameters on MB reduction

#### Catalytic study

**Fig. 4:**
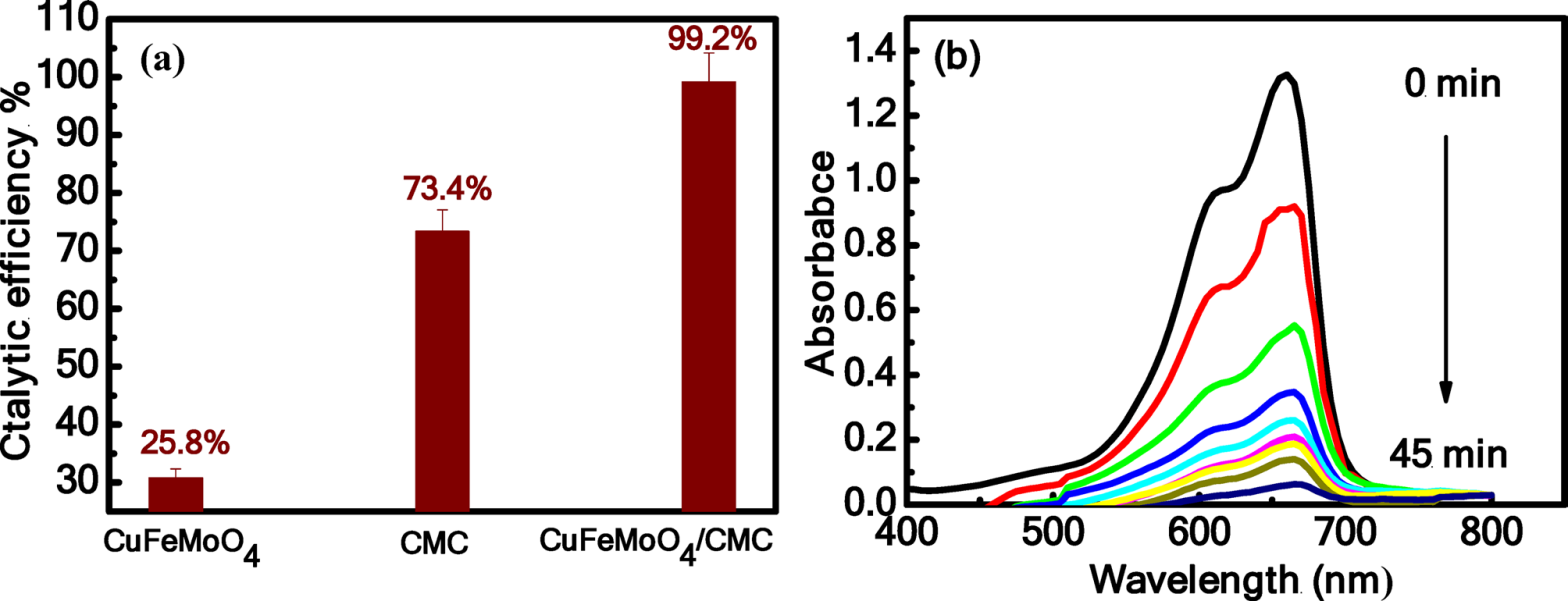
(**a**) Catalytic efficiency of MB reduction as a function of catalyst type, (**b**) The decrease in MB absorbance during the reaction with NaBH_4_ in the presence of CuFeMoO_4_/CMC. Catalyst dose = 0.02 g, [NaBH_4_]_o_ = 41.6 mgL^− 1^, pH = 6, and T = 30 °C.

Based on the reported research, some studies on the reaction of MB with NaBH_4_ showed a very slow reduction reaction, while other studies reported that the dye did not affected and remained stable^44,45^. When the present CuFeMoO_4_/CMC nanocomposite was added to the MB solution in the absence of NaBH_4_ revealed no adsorption over 2 h, indicating that the removal of MB cannot proceed via adsorption. Therefore, it was of interest to do the disconnection of MB from the solution by catalytic reduction in the presence of an efficient catalyst and NaBH_4_. For the sake of comparison, the catalytic reactivity of CuFeMoO_4_/CMC and its individual components (CuFeMoO_4_, CMC) was investigated by introducing these materials into the MB reaction with NaBH_4_. When the kinetics of these reactions were followed, it resulted in a reduction efficiency of the order CuFeMoO_4_/CMC > CMC > CuFeMoO_4_. The pseudo-first-order rate constants also followed the same order CuFeMoO_4_/CMC (0.9609 ± 0.0233 min^−1^) > CMC (0.2274 ± 0.00592 min^−1^) > CuFeMoO_4_ (0.1365 ± 0.00445 min^–1^). The CuFeMoO_4_/CMC exhibited the highest catalytic activity as depicted in Fig. 4(a). The kinetic measurements were carried out by measuring the absorbance decay of MB during the progress of the reaction as observed in Fig. 4(b).

### Effect of catalyst dose

**Fig. 5:**
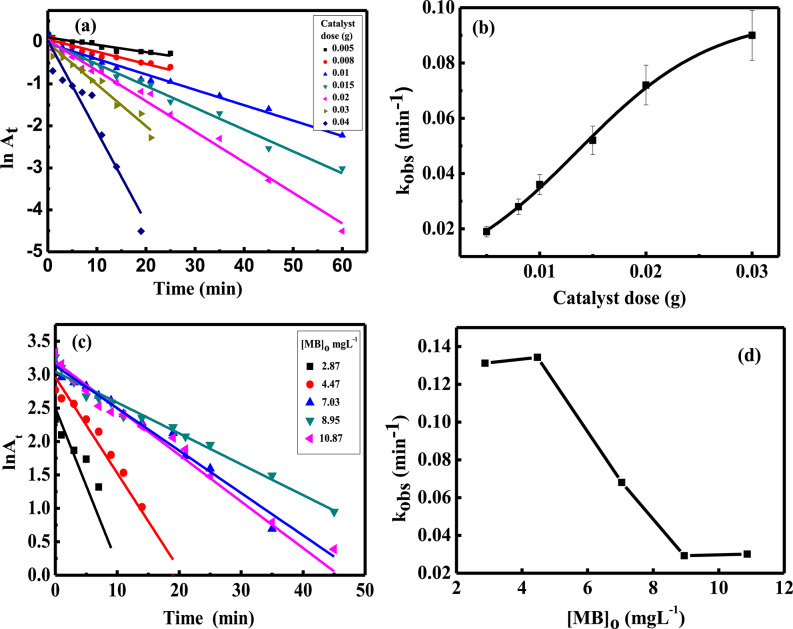
(**a**) First order plots at different dose of CuFeMoO_4_/CMC, (**b**) The rate constant as a function of catalyst dose at [MB]_o_ = 7.03 mgL^− 1^, (**c**) First order plots for the reduction of MB, (**d**) Rate constant as a function of MB concentration at catalyst dose = 0.02 g. [NaBH_4_]_o_ = 41.6 mgL^− 1^, pH = 6, and T = 30 °C.

Changing the catalyst dose is a significant factor in studying the change in the reaction rate. The effect of CuFeMoO_4_/CMC dose on reaction rate was carried out at constant conditions of [MB]_o_ = 7.03 mgL^–1^, pH = 6, [NaBH_4_]_o_ = 41.6 mgL^–1^, and variable catalyst dose ranging from 0.005 to 0.03 g at 30 °C. Regarding the catalyst dose, the reaction under these circumstances followed pseudo-first-order kinetics, Fig. 5(a). The rate constant increased with an increase in the dose from 0.005 to 0.03 g, as seen in Fig. 5(b). The faster removal rate of MB is due to the larger number of active sites available as the composite dosage increases. A higher dosage provides a greater surface area relative to the fixed amount of dye molecules in solution, which in turn favors the adsorption of both MB and NaBH_4_^46^.

### Effect of [MB]_o_

The effect of the initial concentration of MB on the reaction rate was investigated in the concentration range 2.87–10.87 mgL^− 1^, keeping constant the concentration of NaBH_4_ at 41.6 mgL^− 1^, CuFeMoO_4_/CMC dose 0.02 g, pH 6, and temperature 30 °C. Under these conditions, the reaction obeyed pseudo-first order kinetics, ln(A_t_) = ln(A_o_) - kt, with respect to the concentration of MB, Fig. 5(c). The observed rate constant decreased by increasing the initial dye concentration, Fig. 5 (d). The primary factor determining the degradation rate is the number of active sites. With an increase in MB concentration, the available adsorption sites decrease as they get occupied by the existing dye molecules. Consequently, the new molecules can’t adhere to the catalyst surface until the previous ones have degraded.

In addition, a high MB concentration clogs the porous structure of the catalyst and reduces the effective adsorption sites. This impedes the electron transfer process between the reducing agent and the MB, leading to a decrease in the catalytic degradation rate^9^. A further reason that may cause a stepwise decrease in reaction rate, due to the high concentration of the dye, is the possible competition taking place between the reaction products or the generated intermediates and the unreacted dye molecules to interact with the catalyst surface. Such interference of reaction products or intermediates will poison the catalyst surface, leading to an inhibition in reaction rate.

### Effect of [NaBH_4_]_o_

**Fig. 6:**
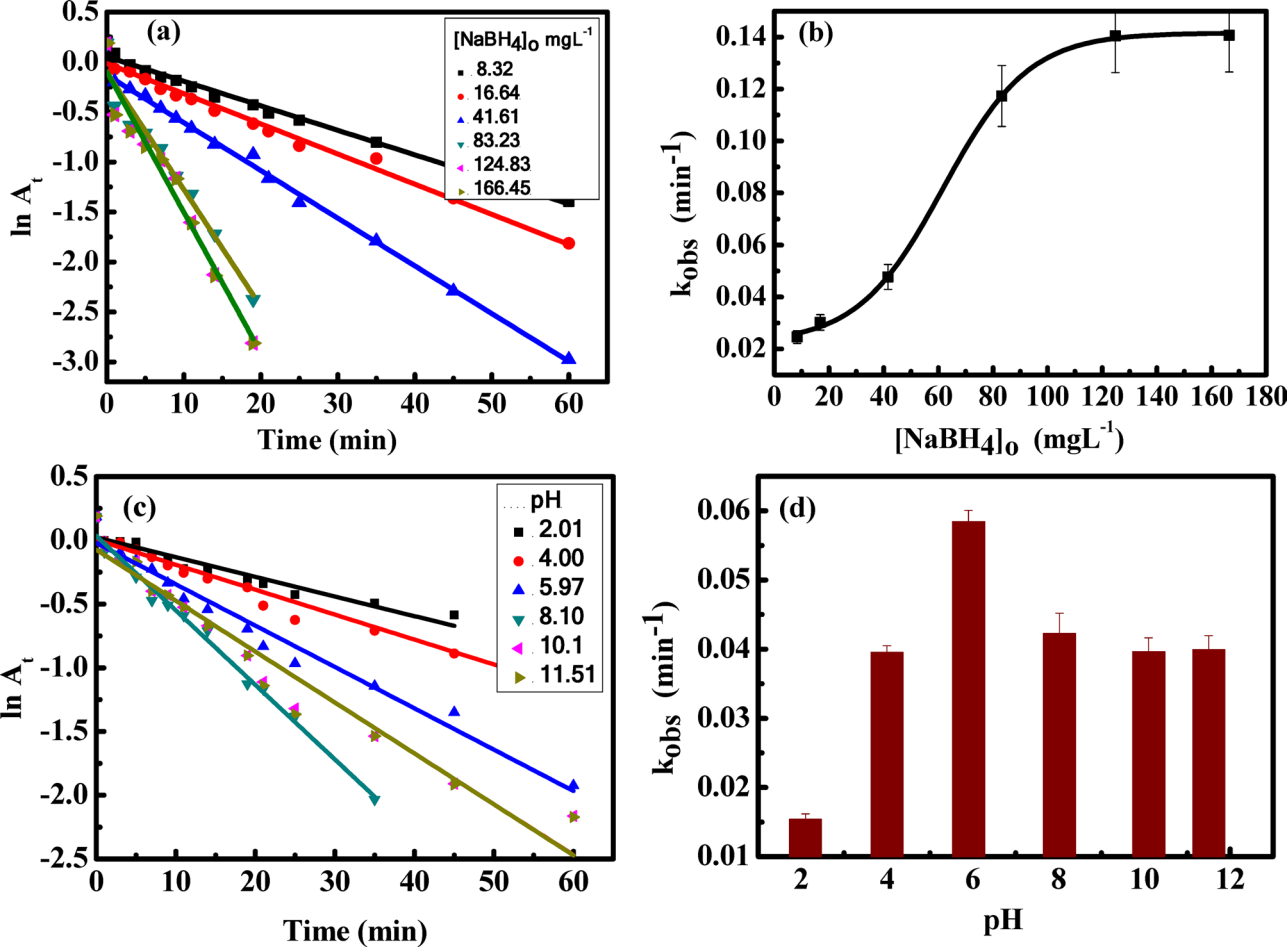
(**a**) First order plots at different concentrations of NaBH_4_, (**b**) Rate constant as a function of NaBH_4_ concentration, (**c**) First order plots for the reduction of MB at variable pH, (**d**) Rate constant as a function of pH. Catalyst dose = 0.02 g, [NaBH_4_]_o_ = 41.6 mgL^− 1^, [MB]_o_ = 7.03 mgL^− 1^, and T = 30 °C.

The effect of NaBH_4_ concentration on the reaction rate was investigated at constant concentration of the dye (7.03 mgL^–1^, fixed amount of the catalyst (0.02 g), pH 6, and 30 °C. The concentration of NaBH_4_ was varied in the range of 8.32–166.4 mgL^–1^. Under these conditions, the reaction obeyed pseudo-first-order kinetics with respect to the concentration of NaBH_4_, Fig. 6 (a). The pseudo-first-order rate constant increased with the increase in the concentration of NaBH_4_, Fig. 6 (b). The degradation of MB was enhanced by the sufficient number of BH_4_^−^ ions generated at higher NaBH_4_ concentrations, which improved the electron transfer process and thus the degradation rate of MB^47^.

### Effect of pH

The solution pH is one of the most important parameters affecting the catalytic reactions. It might affect how the catalyst surface, the dye molecule’s charge is distributed, and how reactive radical species are produced^48^. To find the optimum pH for the present removal of methylene blue, pH buffer solutions of KH_2_PO_4_ and K_2_HPO_4_ were used in the range of 2.1 to 11.5 by adding NaOH or HCl solutions (0.1 × 10^−3^ molL^–1^. The rate of catalytic reduction of MB and degradation percentage are small values in strong acidic conditions (pH 2.1–4). When the pH was raised to 5.9 an increase in catalytic efficiency was observed. The catalytic efficiency increased from 57.16% at pH = 2.1 to 98.8% at pH = 5.9 and then decreased at pH 8, 10.1, and 11.5, Fig. 6 (c, d). The dye structure was also greatly affected by the change of pH, and consequently the removal percentage changed. In an acidic environment (pH < 6), the catalyst’s surface becomes positively charged due to the protonation. This positive charge repels the positively charged methylene blue molecules. The repulsion of both reactants leads to a significant decrease in the reaction rate and the overall efficiency compared to the neutral conditions. On the other hand, under neutral conditions (pH ≈ 6), the catalyst’s surface is generally neutral. This lack of charge allows for optimal adsorption of both the MB and the BH_4_^−^ ions, leading to the highest reaction rate and dye removal percentage. In a basic environment (pH > 6), the deprotonation of hydroxyl (^-^OH) groups on the catalyst’s surface let the surface acquires a negative charge. This negative charge strongly repels the negatively charged BH_4_^−^ ions, significantly reducing their adsorption. Although it may not repel the MB, the reduced availability of BH_4_^−^ ions causes the reaction rate and dye removal efficiency to drop^49,50^.

### Effect of [NaCl]_o_

**Fig. 7:**
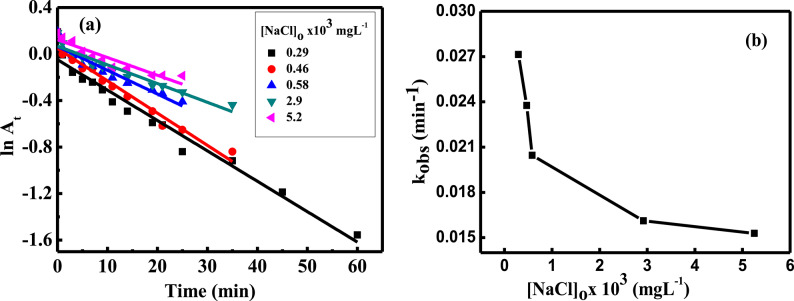
(**a**) First order plots at different concentrations of NaCl, (**b**) Rate constant as a function of NaCl concentration. [MB]_o_ = 7.03 mgL^− 1^, catalyst dose = 0.02 g, [NaBH_4_]_o_ = 41.6 mgL^− 1^, pH = 6, and T = 30 °C.

The presence of salts plays an important role in the dye removal process from environmental effluents. To evaluate the effect of NaCl on the catalytic reduction of MB, various concentrations of NaCl ranging from (0.288 to 2.59) x10^3^ mg L^− 1^ were applied. The reaction rate constant decreased from (0.0261 ± 0.00146) to (0.0161 ± 0.00144) min^− 1^ as the salt concentration increased from 0.3 to 5.25 mg L^− 1^, Fig. 7(a, b). The reduction in the removal rate of MB is due to two main factors: competitive adsorption and the effect of high ionic strength. The catalytic degradation process is highly dependent on the adsorption of both methylene blue molecules and BH_4_^−^ ions onto the catalyst’s active sites. When NaCl is added, it dissociates into Na^+^ and Cl^−^ ions, which are present at a much higher concentration than the reactants. These ions then compete with the MB and BH_4_^−^ for the same active sites on the catalyst’s surface and occupy it. This competition displaces the reactants, significantly decreasing the amount of MB and BH_4_^−^ that can bind. Consequently, the reaction rate decreases because fewer reactant molecules are available to participate in the electron transfer process.

Furthermore, the high concentration of salt ions increases the solution’s ionic strength. This high ionic strength shields the electrostatic charges on the catalyst’s surface and the reactant molecules. This weakens the necessary attraction for binding and hinders the ability of the reactants to adsorb onto the catalyst. The combined effect of the competitive adsorption and this shielding leads to the observed decrease in the dye removal rate^45,51^.

### Effect of temperature

**Fig. 8:**
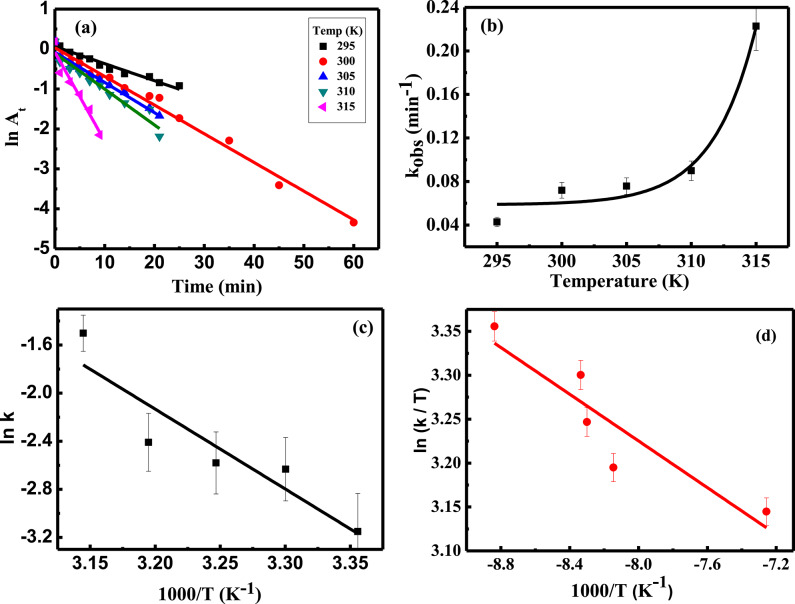
(**a**) First-order kinetic plots at different temperatures, (**b**) Rate constant as a function of temperatures, [MB]_o_ = 7.03 mgL^− 1^, catalyst dose = 0.02 g, [NaBH_4_]_o_ = 41.6 mgL^− 1^, and pH = 6, (**c**) Arrhenius plot, (**d**) Eyring’s plot.

The influence of temperature on MB removal was examined as shown in Fig. 8 (a, b). The rate constant increased as the temperature rose, indicating that the high thermal energy enhances the mobility of both borohydride ions (BH₄⁻) and dye molecules, thereby increasing collision frequency and improving their interaction with the catalyst’s active sites. From the Arrhenius plot of ln k vs. 1/T (Fig. 8c), the activation energy (Eₐ) and other activation parameters were determined, Table 1. The positive value of ΔH^#^ (52.67 kJmol^−1^, confirms the endothermic nature of the reduction process. The negative ΔS^#^ (− 95.93 Jmol^−1^ K^−1^ reflects increased randomness at the solid/solution interface, while the positive ΔG^#^ (82.21 kJ/mol^−1^) indicates that the catalytic reduction of MB is non-spontaneous under the studied conditions^52^. The thermodynamic parameters provide essential insights into the energy changes that occurred during the catalytic process. They were evaluated using the Eyring plot (Fig. 8d). The obtained values (Table 2) reveal a positive ΔH° (11.04 kJmol^−1^, suggesting endothermic reduction of MB. The negative ΔS° (− 179.48 Jmol^-1^ K^-1^ implies high disorder of the catalytic reaction. The positive ΔG° (55.28 kJmol^−1^ confirms that MB adsorption is also non-spontaneous^53,54^.

**Table 1:** Activation parameters of MB degradation, [MB]_o_ = 7.03 mgL^− 1^, [NaBH_4_]_o_ = 41.6 mgL^− 1^, pH = 6, and catalyst dose = 0.02 g.

Temp	k	E_a_	∆H^#^	∆G^#^	∆S^#^
k	min^− 1^	kJmol^− 1^	kJmol^− 1^	kJmol^− 1^	Jmol^−1^K^− 1^
298	0.0428 (± 0.002)				
303	0.0718 (± 0.002)				
308	0.0757 (± 0.005)	55.23	52.67	82.21	−95.93
313	0.0898 (± 0.008)				
318	0.2227 (± 0.027)				

**Table 2:** Thermodynamic parameters of MB degradation, [MB]_o_ = 7.03 mgL^− 1^, [NaBH_4_]_o_ = 41.6 mgL^− 1^, pH = 6 and catalyst dose = 0.02 g.

Temp	k	∆H^o^	∆G^o^	∆S^o^
K	min^− 1^	kJmol^− 1^	kJmol^− 1^	Jmol^−1^K^− 1^
298	0.0428 (± 0.002)			
303	0.0718 (± 0.002)			
308	0.0757 (± 0.005)	11.04	55.28	−179.48
313	0.0898 (± 0.008)			
318	0.2227 (± 0.027)			

#### Effect of a radical scavenger

**Fig. 9:**
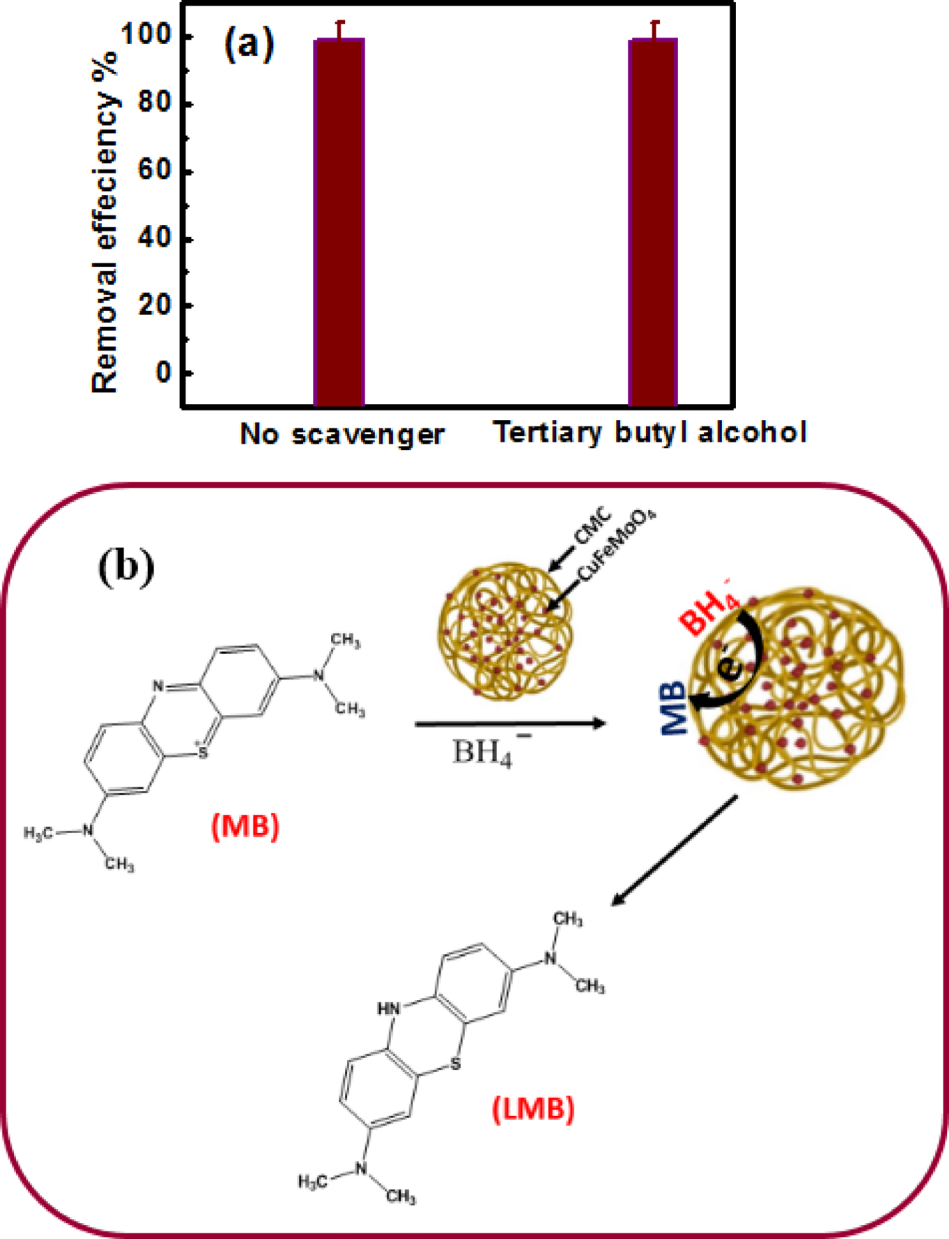
(**a**) The role of radical scavenger (**b**) A proposed mechanism of MB reduction by NaBH_4_ in the presence of CuFeMoO_4_/CMC as catalyst.

The tertiary butyl alcohol (TBA) is known to capture a variety of radical species. In order to investigate if a radical species is involved in the current catalytic reduction of MB with NaBH_4_, it has been employed as a radical scavenger at an initial concentration of 0.048 mol L^− 1^. Both with and without tertiary butyl alcohol, Fig. 9 (a) shows how the dye’s absorbance decreases with time. The dye absorbance remained constant when the tertiary butyl alcohol is present in the solution, suggesting that the intermediate radicals are not involved in the reduction of MB with NaBH₄ in the presence of the nanocomposite^55,56^. The blue MB changed to the colorless leucomethylene blue (LMB) via electron transfer from NaBH_4_ to the catalyst and then to the MB molecules.

### Mechanism of MB reduction

The reduction of MB by NaBH_4_ did not occur in the absence of the CuFeMoO_4_/CMC catalyst. In the presence of the catalyst, MB was reduced to the colorless leucomethylene blue (LMB) via electron transfer from NaBH_4_ to the catalyst surface and then to the MB molecules. Such an electron-shuttling surface was illustrated by the Langmuir-Hinshelwood model^57^ The NaBH_4_ is acting as an electron donor and hydrogen supplier. It dissociates into Na^+^ and BH_4_^−^ ions. The BH_4_^−^ ions donate electrons to the surface of CuFeMoO_4_/CMC, which in turn transfer these electrons to the MB to start the reduction process at the catalyst surface. On the other hand, the MB is a cationic dye that belongs to the thiazine class of dyes, where the hydrogen species generated from the BH_4_^−^ ions attack the thiazine group of the MB molecule following the electron transfer to the CuFeMoO_4_/CMC surface^58^. This reaction leads to the formation of the colorless leucomethylene blue form of the MB. The synergistic effect between CuO, FeO, and MoO_4_ speeds up the rate of electron transfer and enhances the reduction process. Figure. 9 (b) illustrates a proposed mechanism of MB reduction by NaBH_4_ in the presence of CuFeMoO_4_/CMC as a catalyst^59^. The following redox mechanism depicts how the Cu, Fe, and Mo contribute to the electron transfer process.

$$B{H_4}^ -\rightarrow {e^ - }\rightarrow\left( {C{u^{2 + }},{\text{ }}F{e^{3 + }},{\text{ }}M{o^{6 + }}} \right)\;\rightarrow\left( {C{u^ + },{\text{ }}F{e^{2 + }},{\text{ }}M{o^{5 + }}} \right)$$

$$\left( {C{u^ + },{\text{ }}F{e^{2 + }},{\text{ }}M{o^{5 + }}} \right)\;\rightarrow{e^ - }\rightarrow MB\rightarrow LMB{\text{ }} + {\text{ }}\left( {C{u^{2 + }},{\text{ }}F{e^{3 + }},{\text{ }}M{o^{6 + }}} \right)$$

 

The Cu^2+^, Fe^3+^, and Mo^6+^ centers in the CuFeMoO_4_/CMC catalyst undergo reduction through an electron transfer from BH_4_^−^, yielding the corresponding lower-valent species (Cu^+^, Fe^2+^, Mo^5+^). These reduced metal ions subsequently act as electron mediators, delivering the acquired electrons to MB converting it into the leucomethylene blue (LMB). Following this electron donation, the metal ion centers revert to their oxidized states (Cu^2+^, Fe^3+^, Mo^6+^), sustaining continuous turnover of the catalytic redox cycle. The following reactions illustrate further the catalytic interactions between MB and NaBH_4_.

2 $$MB{\text{ }} + {\text{ }}S\;\; \rightleftharpoons \;\;MB - S$$

3 $$B{H_4}^ - + {\text{ }}S\;\; \rightleftharpoons \;\;B{H_4}^ - - S$$

4 $$MB-S{\text{ }} + {\text{ }}B{H_4}^--S\rightarrow\;LMB-S{\text{ }} + {\text{ }}S$$

5 $$LMB - S\;\;\; \rightleftharpoons \;\;\;\;LMB{\text{ }} + {\text{ }}S$$

where S represents the free active site on the CuFeMoO_4_/CMC surface, MB-S for adsorbed MB, BH_4_^−^-S for adsorbed BH_4_^−^, and LMB-S for the formed LMB on the surface, and LMB for the desorbed leucomethylene blue.

### Regeneration of CuFeMoO_4_/CMC

**Fig. 10:**
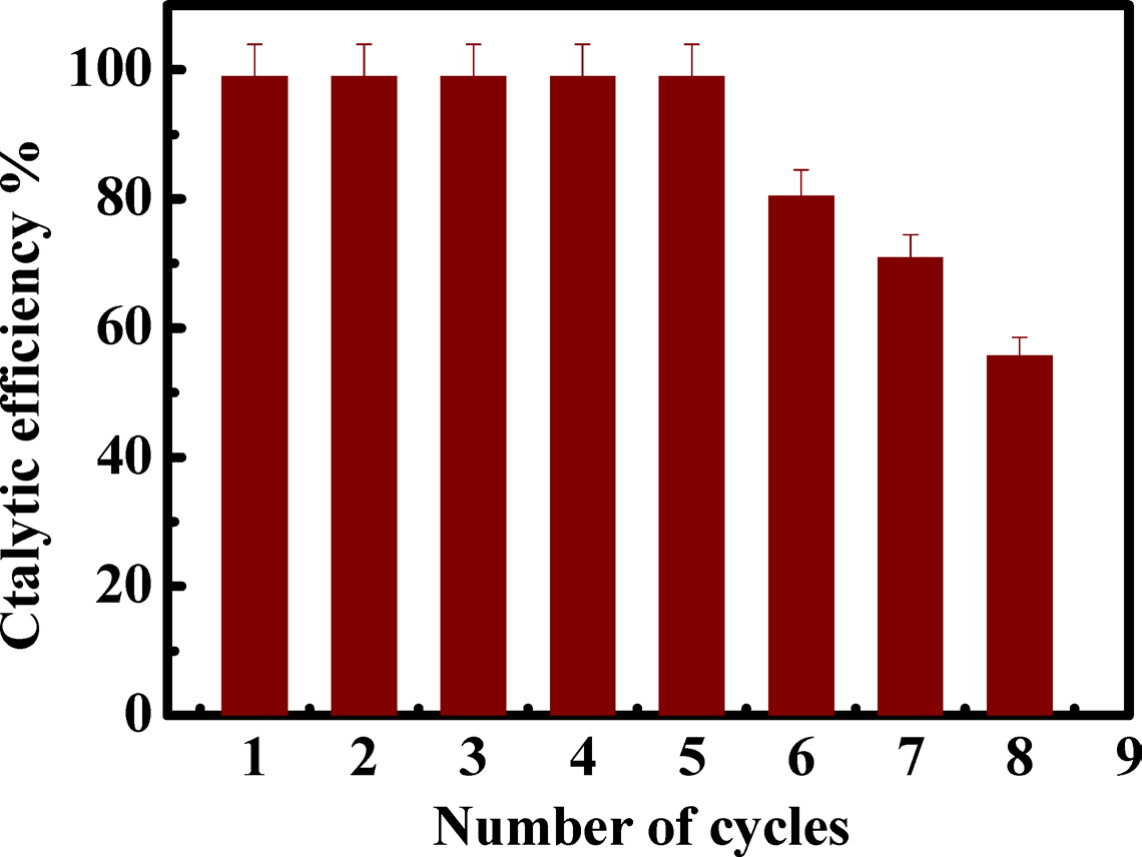
The reusability of CuFeMoO_4_/CMC in MB removal from solution, [MB]_o_ = 7.03 mgL^− 1^, catalyst dose = 0.02 g, [NaBH_4_]_o_ = 41.6 mgL^− 1^, pH = 6, and T = 30 °C.

**Fig. 11:**
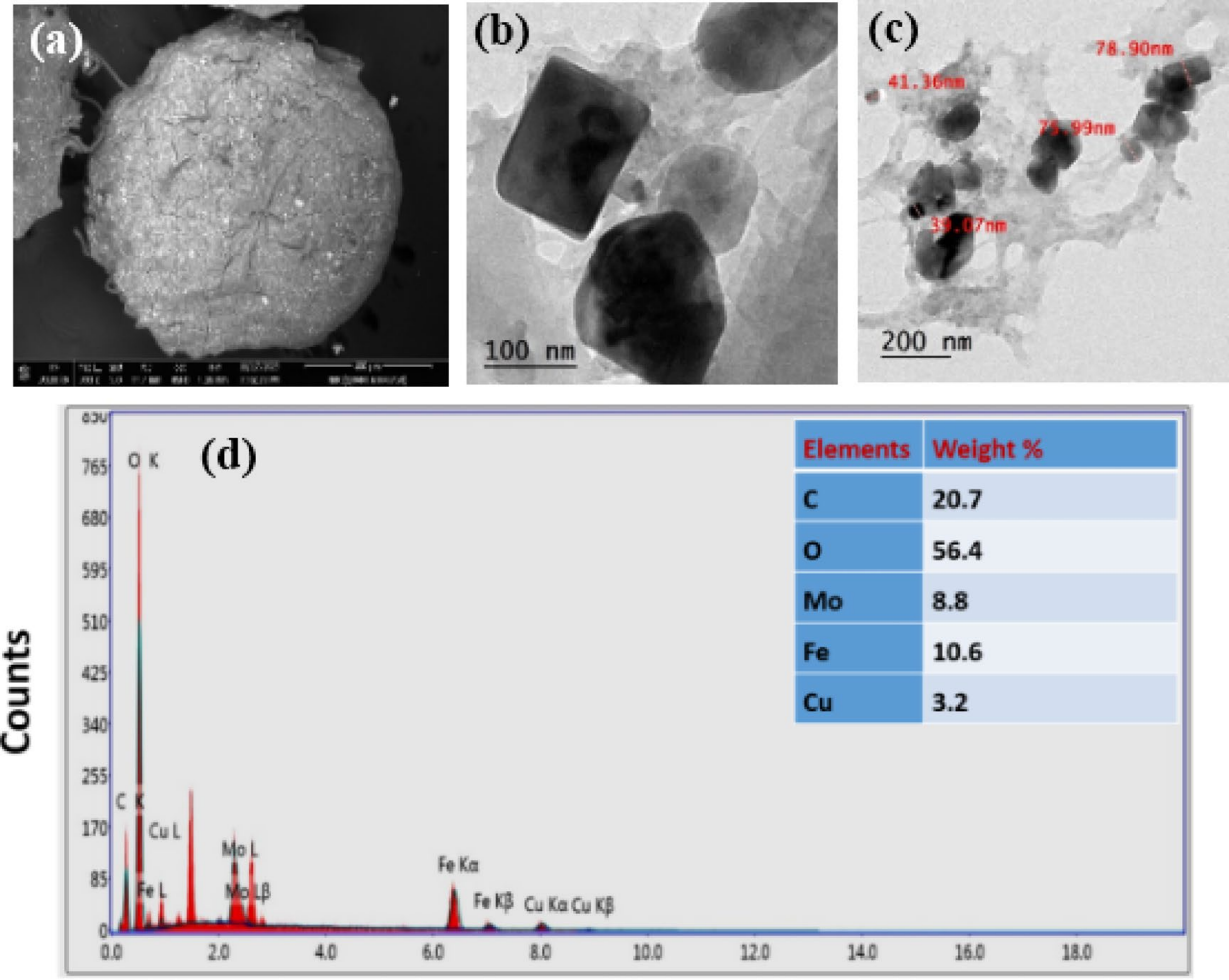
(**a**) SEM micrographs, (**b**& **c**) TEM micrographs, (**d**) EDX analysis of CuFeMoO_4_/CMC after MB reduction.

From an economic perspective, catalyst stability is considered a crucial factor for the application. After the first run of methylene blue reduction using the microbeads as catalysts, the catalyst was filtered, washed several times with water, and dried at 30 °C. The regenerated catalyst was added to a fresh methylene blue solution, and the degradation run was performed under the optimum conditions, pH 6, catalyst dose 0.02 g, [MB]_o_ = 7.03 mgL^− 1^, [NaBH_4_]_o_ = 41.6 mgL^− 1^ at 30 °C. The degradation-regeneration process was repeated up to eight cycles. No decay was observed in the catalytic activity of the catalyst up to the fifth cycle. Beyond the fifth cycle, the activity decreased from 99 to 55.8%, confirming that the CuFeMoO_4_/CMC is a highly recyclable, reusable, and effective catalyst for the MB removal from wastewater, Fig. 10. The post-reduction characterization reveals significant morphological and structural changes in the CuFeMoO_4_/CMC microbeads surface. The SEM micrograph in Fig. 11(a) illustrates that the microbeads largely maintained their original spherical shape with some roughness and fracture compared to the pre-MB reduction state. The TEM micrographs in Fig. 11 (b & c) show a clear morphological change in the embedded nanoparticles. The embedded nanoparticles appear in irregular or near-cubic shapes and exhibit noticeable aggregation with specific size measurements, indicating the dimensions of the nanoparticles within these clusters. It also reveals the persistence of well-defined, discrete nanoparticles, indicating the successful preservation of the active structure. From a compositional perspective, the EDX analysis in Fig. 11(d) confirms the continued presence of all constituent elements (C, O, Mo, Fe, Cu). Although the relative ratio of the metallic elements appears high, the observed morphological transformation is consistent with a chemical change (reduction) that typically involves a reduction in the overall oxygen content (O wt%). This high degree of elemental retention validates the exceptional chemical stability and reusability of the CuFeMoO_4_/CMC nanocatalyst, making it highly suitable for sustainable catalytic applications.

### Comparative catalytic study

The main objective of this study was to create a unique nanocomposite, such as the CuFeMoO_4_/CMC nanocomposite, that would be able to extract the MB from water more efficiently than previous nanocomposites reported in the literature. Table 3 lists the studies conducted on the removal of MB by different catalysts. Given the wide range of experimental conditions used in prior studies, the present study demonstrates a suitable rate constant of 0.9609 ± 0.0233 min^−1^ and a higher efficiency of 99% in a shorter time of 45 min^10,45,60–66^.

**Table 3:** A comparison of the nanomaterial’s catalytic efficiency and rate constant of MB activity.

Catalyst	Catalytic Efficiency %	Rate constant (min^− 1^)	Refs
Ag_2_MoO_4_/BiOBr Photocatalyst	93.8%	0.086	10
Fe_3_O_4_/SiO_2_ nanoparticles	99.8%	0.522	43
Novel copper nickel oxysulfide (CuNiOS)	75%	0.144	54
Fe_3_O_4_@SiO_2_/Ag	96.5%	0.091	55
Fe_3_O_4_@Tannic acid@Au	90%	0.441	56
CuMoO_4_/ZnO nanocomposites	92%	0.359	57
Co-magnetic nanoparticles	85.4%	0.049	58
Alginate@Fe_3_O_4_ beads	88%	0.012	59
Ag/Fe_3_O_4_@GO nanoparticles	97%	0.448	[60
CuFeMoO_4_/CMC microbeads	99%	0.960	Present study

### Computational study

The MB dye was subjected to the Density Functional Theory (DFT) computations in both gas and aqueous phases using Gaussian 09 software, the B3LYP technique, and the 6–311 + + G(d, p) basis set^67^. This approach has become more well-known in recent years since it can yield precise findings in a comparatively short amount of time. Data visualization was carried out with Gaussian View 5.0^68^. The gap energy (ΔE), electronegativity (χ), proportion of charge transfer (ΔN), electron affinity (Gap energy (ΔE), electronegativity (χ), proportion of charge transfer (ΔN), electron affinity (EA), ionization potential (IP), dipole moment, E_HOMO_, E_LUMO_, and other computational parameters such as global softness (σ), global hardness (η), electrophilicity (ω), and chemical potential (µ) were calculated in both gas phase and water solvent. Furthermore, the molecular electrostatic potential (MEP), natural bonding orbital (NBO), FT-IR, and UV-time dependent calculations were performed using the DFT 6-31G++ (d, p) method. The solvent effect (IEFPCM) on molecular and electronic structures was represented using the polarizable model formalism.

6 $$\Delta E{\text{ }} = {\text{ }}{E_{LUMO}}-{\text{ }}{E_{HOMO}}$$

7 $$\chi {\text{ }} = {\text{ }} - \mu= \; -\frac{{E}_{HOMO}+{E}_{LUMO}}{2}$$

8 $$\eta= -\frac{{E}_{HOMO}-{E}_{LUMO}}{2}$$

9 $$\sigma=\frac{1}{\eta}$$

10 $$\Delta N=-\frac{\mu}{\eta}$$

11 $$EA = {\text{ }} - {\text{ }}LUMO$$

12 $$IP{\text{ }} = {\text{ }} - {\text{ }}HOMO$$

13 $$\omega {\text{ }} = {\text{ }}{\mu ^2}/2\eta$$

### Quantum chemical calculations

**Fig. 12:**
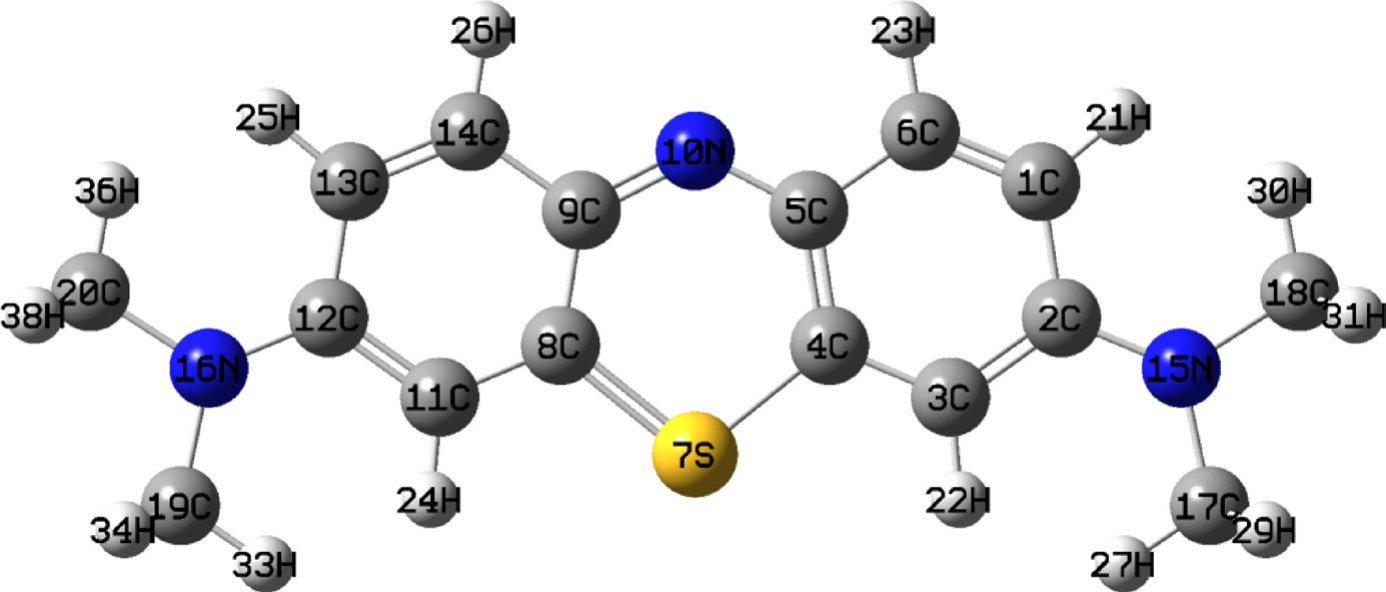
Optimized geometrical structure of MB obtained from the DFT calculations.

The calculated quantum chemical descriptors provide trends in the reactivity and selectivity features of the studied MB dye. Using the DFT theory and basis functions 6-31G (++) (d, p), structural parameters, geometry optimization, and time dependent-DFT (TD-DFT) for the MB were determined. A review of the literature revealed that methylene blue was utilized in a study where a positive charge appeared on the N-atom and occasionally on the S-atom, but no one identified which one was in charge of MB’s reactivity. In order to comprehend and validate the mechanism of MB degradation, we began by demonstrating which structure is more reactive. From the calculated quantum chemical descriptors mentioned in Table 4, the MB with positive charge on S-atom has higher HOMO (−10.206 eV), electron affinity (6.131 eV), and electrophilicity (16.378 eV), lower LUMO (−6.131 eV), ∆E (4.075 eV), with lower dipole moment (1.873 D), electronegativity (8.168 e), and ∆N (2.532 e). This indicates that MB that has a positive charge on the S-atom is more reactive than one that has a positive charge on the N-atom. Therefore, we anticipate that the most favorable electrophilic site for adsorption by the nanocomposite is the positively charged sulfur atom in the MB molecule. As a result, we continued the theoretical study on the MB with the positive charge on the S-atom. The optimized structure of the MB given in Fig. 12 was examined using the DFT 6-31G++ (d.p).

**Table 4:** The quantum chemical parameters calculated for MB with positive S- and N-atoms using DFT/6-31G++ (d, p).

Parameter	MB (positive S)	MB (positive *N*)
E_HOMO_, Ev	−10.206	−12.464
E_LUMO_, eV	−6.131	−5.406
ΔE, eV	4.075	7.057
Dipole moment	1.873	1.906
Global hardness (η), eV	2.037	3.528
Global softness (σ), eV^− 1^	0.490	0.283
Ionization potential (IP)	10.206	12.464
Electron affinity (EA)	6.131	5.406
Electronegativity (χ), eV	8.168	8.935
Chemical potential(µ), eV	−8.168	−8.935
Electrophilicity (ω)	16.378	11.314
ΔN_max_ (e)	2.532	4.009

**Fig. 13:**
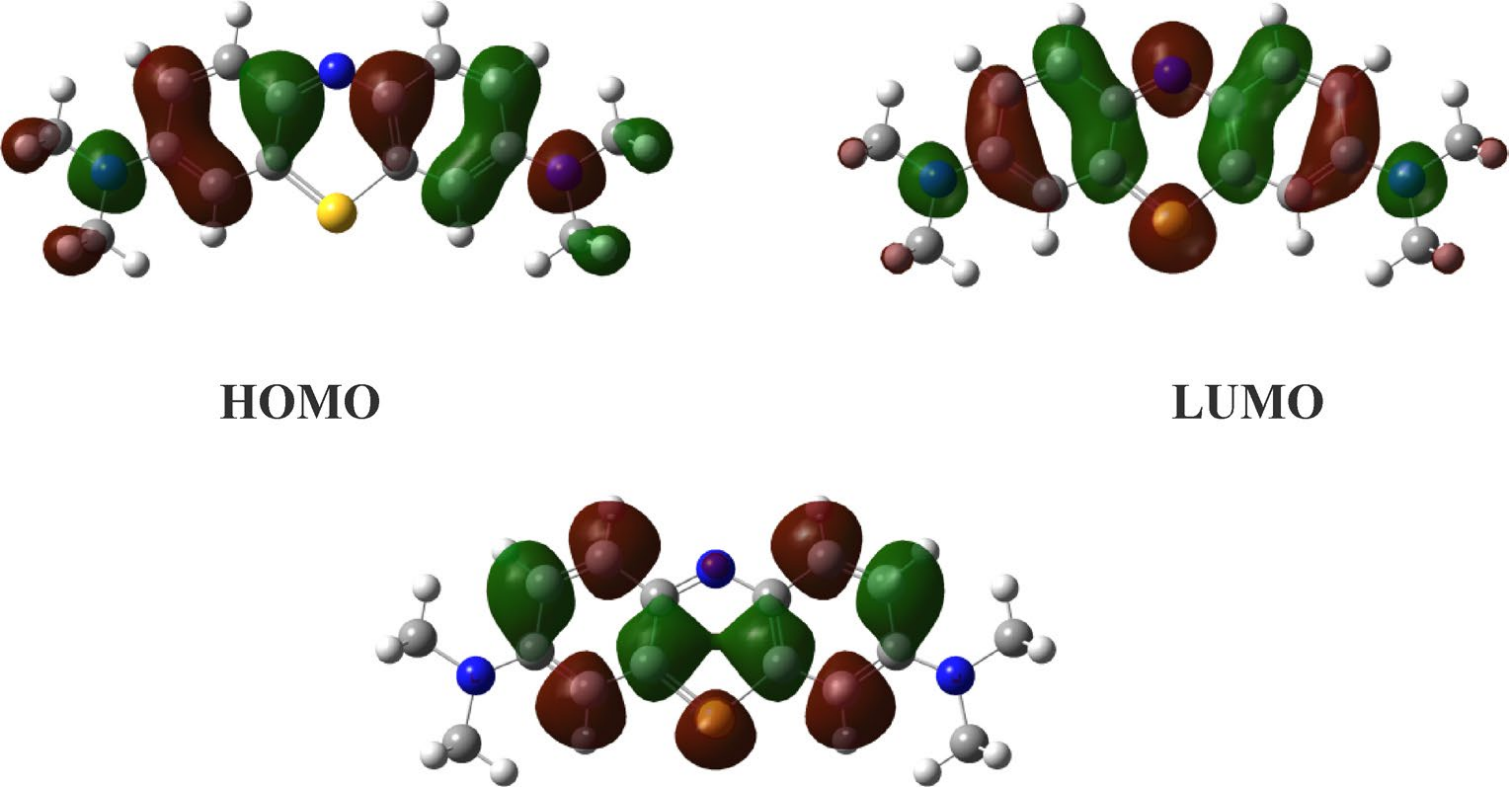
Frontier molecular orbitals, HOMO, LUMO, and LUMO + 2 in water solvent.

DFT’s quantum chemical parameters were utilized to comprehend how the nanocomposite and MB interacted. It is obvious from Table 5 that the highest occupied and lowest unoccupied molecular orbitals demonstrated a significant influence in molecular interaction. Firstly, the IP and the HOMO level are intimately connected. A higher EHOMO value indicates that MB is more efficient since it can donate electrons to the surface of the nanocomposite. The calculations in Table 5 demonstrated that the water solvent’s effect resulted in an increase in the HOMO values, which rose up from − 10.206 to −8.045 eV, in gas phase and water, respectively. This could imply that the MB is more reactive to the water-phase nanocomposite than to the gas-phase. The LUMO value and EA also have a direct relationship. The molecule’s capacity to absorb additional electrons from the nanocomposite would therefore be improved by E_LUMO_ from − 6.131 to −3.467 eV, in both gas and water, respectively. This entails making MB less reactive in the water solvent so that it may absorb electron density from the nanocomposite. The energy needed to move a charge from the HOMO to the LUMO orbitals is called the gap energy (ΔE). Therefore, the reactivity of MB increases when the energy gap decreases. According to the computations, the water phase’s ΔE values climbed from 4.075 eV in the gas phase to 4.578 eV. This entails lowering the solvent’s dye reactivity with respect to the gas phase. One of the most important factors is the dipole moment, which assesses the polarity of a polar covalent bond; an increase in the dipole moment raises the reactivity. The dipole moment in the water solvent was found to be 2.738 D, which is greater than that in the gas phase (1.873 D). This indicates that the dye reactivity in the water solvent was higher towards the nanocomposite. To assess the chemical reactivity of MB, the chemical potential (µ) and electronegativity (χ) were calculated. The MB’s electronegativity dropped from 8.168 eV in the gas phase to 5.756 eV in the aqueous solvent, and the chemical potential (µ) increased. This could be the cause of the MB’s lower reactivity when it came into contact with the nanocomposite. On the other side, global softness and global hardness have a major role in reactivity prediction, with the hard molecule being less reactive than the soft one. Reducing the reactivity of MB in aqueous solvent, its global softness in the solvent was found to be 0.436 eV as opposed to 0.490 eV in the gas phase. The affinity-giving electrons in the dye is represented by the fraction of charge transfer. The greater the ΔN value, the higher the probability that the dye will add charges to the nanocomposite. Indicating that the dye’s capacity to absorb electrons from CuFeMoO_4_/CMC and NaBH_4_ improved in the water solvent, the value of ΔN dropped from 2.532 e in the gas phase to 2.514 e in the solvent^69^. MB is a cationic dye that is derived from phenothiazine. Figure 13 displays the HOMO and LUMO charge density distributions of MB. All of the MB dye’s skeleton, with the exception of the positive S, is fully localized in terms of the HOMO electron density arrangement. The localized π* character in LUMO is over the positive S-atom. The S-atom is the center of the entire spatial distribution of MB’s energy-varying LUMO + 2 electron density. Therefore, the LUMO + 2 dominates the charge density for electrophilic activity. As a result, it is predicted that sulfur will adsorb the dye molecule more effectively than the nitrogen atom of the benzene ring.

**Table 5:** The quantum chemical parameters calculated for MB in gas phase and water solvent using DFT/6-31G++ (d, p).

Parameters	Gas phase	Water solvent
E_HOMO_, eV	−10.206	−8.045
E_LUMO_, eV	−6.131	−3.467
ΔE, eV	4.075	4.578
Dipole moment	1.873	2.738
Global hardness (η), eV	2.037	2.289
Global softness (σ), eV^− 1^	0.490	0.436
Ionization potential (IP)	10.206	8.045
Electron affinity (EA)	6.131	3.467
Electronegativity (χ), eV	8.168	5.756
Chemical potential(µ), eV	−8.168	−5.756
Electrophilicity (ω)	16.378	7.237
ΔN_max_ (e)	2.532	2.514

### Molecular electrostatic potential

**Fig. 14:**
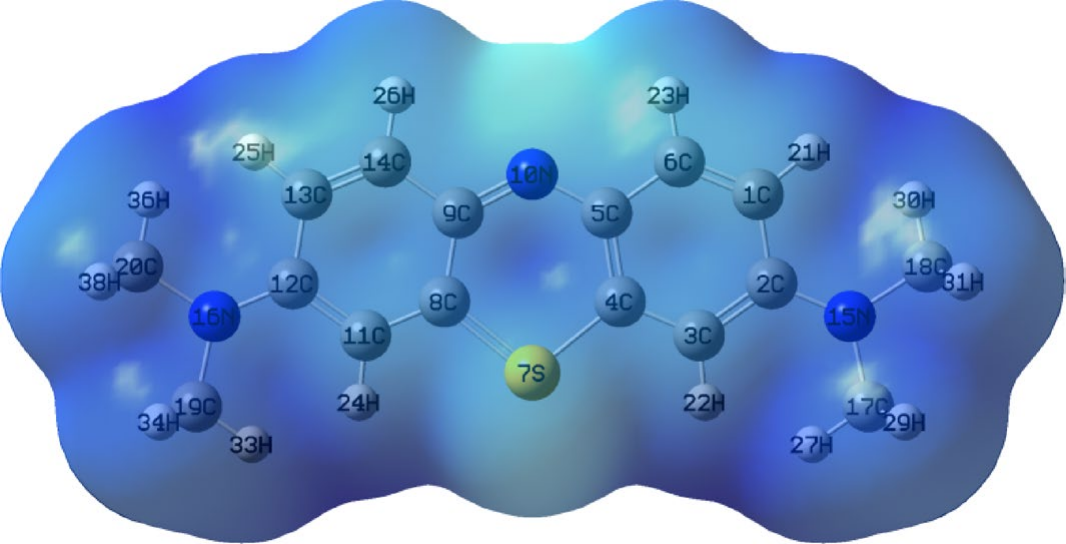
Molecular electrostatic potential (MEP) of MB obtained from DFT calculations.

**Fig. 15:**
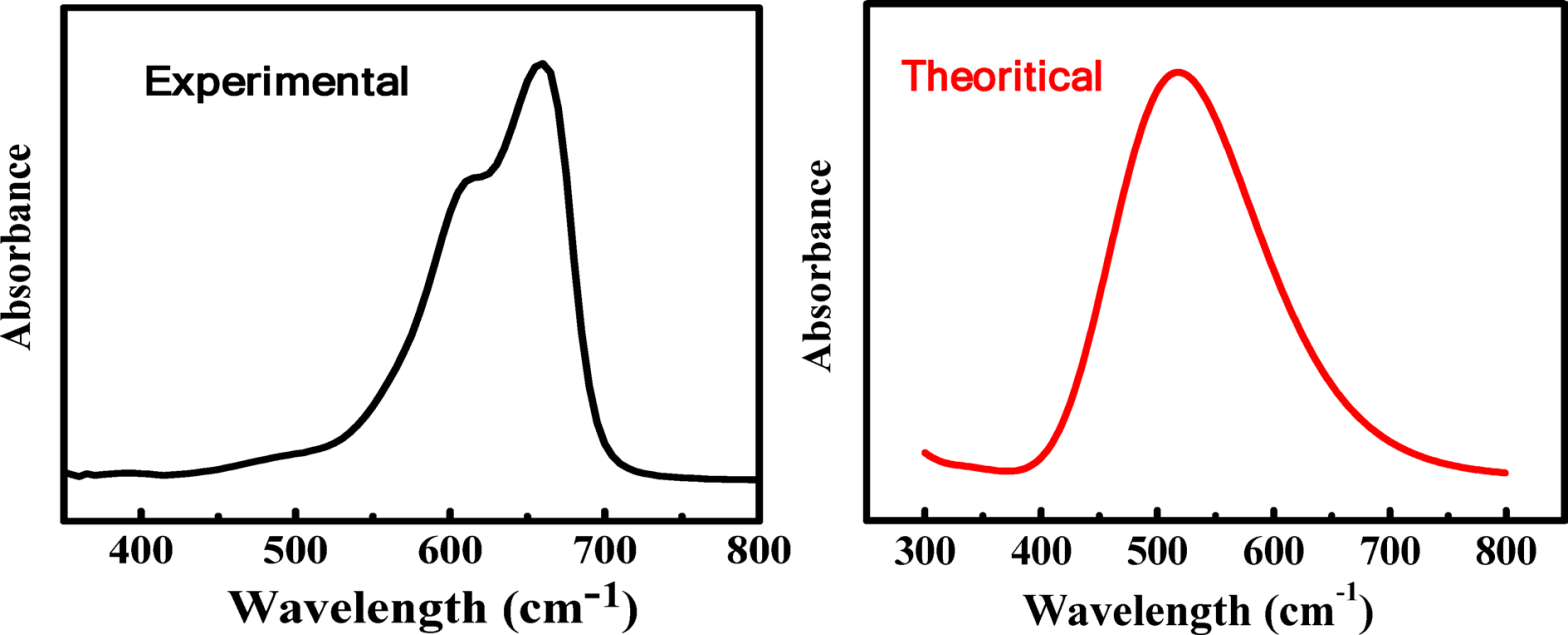
Experimental and computational UV/Vis spectra of MB.

The MEP map can be used as a model to study the polarity of molecules. The relative positions of electrophilic, nucleophilic sites, and zero electrostatic potential regions were ascertained using it^70^. The electrostatic potential allows the potential of the nearby atoms to interact with one another; the MEP is shown in Fig. 14. The blue positive areas are linked to electrophilic reactivity, whereas the red negative zones are linked to nucleophilic activity. Strong electrophilic centering of the sulfur atom in the MB structure predicted a good site for adsorption onto the CuFeMoO_4_/CMC surface. The positive charge on S-atom makes the dye appears more electrophilic, while the sulfur atom gave the dye its blue color, Fig. 15. This indicates the best possibility for the sulfur atom to absorb electrons from the nancomposite surface^30^.

#### UV-Vis and TD-DFT calculations

**Fig. 16:**
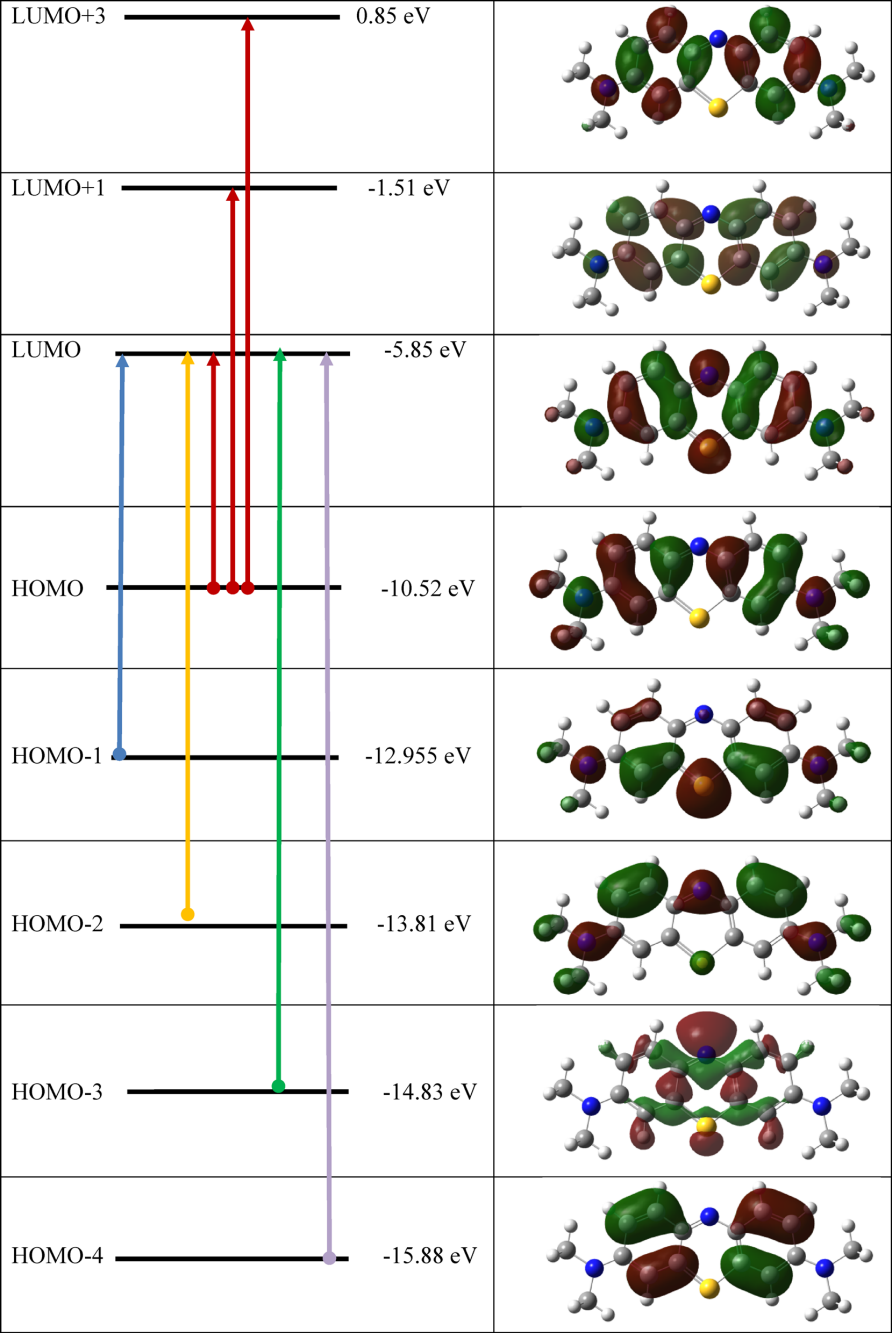
MB molecular orbital diagram in water solvent with electronic excitation obtained from TD-DFT.

A straightforward, safe analytical method that offers vital information on the fundamental electronic transitions in organic molecules is the UV-Vis spectroscopy. To gain a better understanding of the observed spectrum, single-point computations for several singlet excited states are performed using the TD-DFT. We only describe the states with the strongest oscillation. The computational descriptors from TD-DFT are displayed in Table 6, where λ_max_ stands for excitation wavelength, E for excitation energy, and $f$ for oscillator strength. Moreover, a more thorough examination of the computational data might yield vital details to validate the outcomes through experimentation. Both computational and experimental studies on the UV-Vis of MB revealed a good agreement. There is only one absorption band during the experimental work, which indicates the $\mathrm{n}\to{{\uppi}}^{\mathrm{*}}$transition. The bands consist of multiple transitions; some of these transitions come from the π characters, while others come from nonbonding characters, Fig. 15. In Fig. 16, the molecular orbital diagram, which shows the most important electronic transitions between various molecular orbitals, supports this interpretation. The computed and measured wavelengths for the first transition, representing one transition from HOMO to LUMO with an oscillator strength of 1.159, coincide well. This leads us to conclude that the $\mathrm{n}\to{{\uppi}}^{\mathrm{*}}$ transition makes up the transition at 530.8 nm. Moving onto the second transition with the highest contribution, this transition is primarily composed of HOMO-1 to LUMO with an oscillator strength of 0.003. Table 6 displays the computational descriptors for the MB transitions. It is composed of many transitions, some from the nonbonding character and some from the π character, and it correlated well with the UV spectrum recorded experimentally. Analysis of the TD-DFT calculations and the resulting data show a strong correlation with the experimental data.

**Table 6:** The TD-DFT descriptors for the MB in water solvent with the experimental between the bracket.

Excited state	λ (nm)	E (eV)	(f)	Contribution		Transition
1	530.6 665(exp)	2.393	1.159	75 → 76	100%	HOMO → LUMO
2	463.9	2.672	0.003	72 → 76	3.7%	HOMO-3 → LUMO
				74 → 76	95.6%	HOMO-1 → LUMO
3	393.5	3.150	0.002	73 → 76	99.5%	HOMO-2 → LUMO
4	334.3	3.708	0.027	72 → 76	85.6%	HOMO-3 → LUMO
				75 → 77	2.7%	HOMO → LUMO + 1
				75 → 79	8.1%	HOMO → LUMO + 3
5	321.5	3.856	0.001	71 → 76	98.9%	HOMO-4 → LUMO
6	280.7	4.416	0.102	72 → 76	3.9%	HOMO-3 → LUMO
				75 → 77	78.5%	HOMO → LUMO + 1
				75 → 79	14.3%	HOMO → LUMO + 3

### Natural bonding orbital (NBO) analysis

In the molecular system, the NBO analysis appears to be a useful technique for investigating conjugative contacts, charge transfer, interactions in both occupied and vacant orbital regions, and intramolecular and intermolecular interactions^71^. A measure of intramolecular delocalization, the B3LYP/6-31G ++ (d, p) level, was utilized to explain different second-order interactions between the filled orbitals of one subsystem and the vacant orbitals of another subsystem. The stabilizing energy E(2) related to electron delocalization i→j was calculated for each donor (i) and acceptor (j) as follows:

14 $$E(2) = \Delta Eij = qi\frac{{{{(Fij)}^2}}}{{(Ei - Ej)}}$$

where the orbital occupancy and diagonal elements are qi, Ei, and Ej, respectively, and the off-diagonal Fock matrix member is Fij. The ability of electrons to contribute, the strength of the interaction between electron donors and acceptors, and the overall system’s degree of conjugation all increase with the stabilizing energy’s E(2) value. The likely potent interaction of MB is shown in Table 7. Strong intra-molecular hyper-conjugative interactions of π-electrons were found when the Fock matrix in NBO was investigated using the second-order perturbation theory, which led to system stability. Hyper-conjugative interactions reversed of π (C8-C11) → π*(C9-N10) and π (C9-N10) → π*(C8-C11), π (C9-N10) → π*(C13-C14) and π (C13-C14) → π*(C9-N10) are responsible for benzene ring bond conjugation. Moreover, electron donation is one of the most important interactions linked to thiazine ring resonance. From LP (1) C5→ π*(C9-N10) with E (2) equals 90.03 kJ/mol, and the LP (1) N10→ σ*(C4-C5)/σ*(C8-C9) with value of 12.26 kJ/mol for both. And LP (2) S7→ π*(C3-C4)/π*(C8-C11) with E(2) value 19.28 kJ/mol for both. The n and π* bond orbitals overlapped to create the intramolecular hyperconjugative interactions, which led to intramolecular charge transfer (ICT). This rendered the system to stabilize from LP (2) S7→σ*(C3-C4), which raised the ED (0.453e) and weakened the corresponding bonds, to give a high stabilization energy of 19.28 kJmol^− 1^. Furthermore, LP (1) C5→π*(C9-N10) increases the ED (0.358e) and weakens the corresponding bonds, resulting in a strong stabilization (90.03 kJ mol^− 1^)^72^.

**Table 7:** Analysis of the Fock matrix in NBO basis using second order perturbation theory, related to the intramolecular bonds of MB dye.

Donor NBO	Type	ED/e	Acceptor NBO	Type	ED/e	E(2) KJ/mol	E(j)-E(i) a.u	E(j)-E(i) a.u
C1-C2	σ	1.515	N15	*π	0.331	1.20	2.09	0.045
			C1-C6	*σ	0.451	2.66	1.31	0.053
			C2-C3	*σ	0.025	2.79	1.20	0.052
			N15-C17	*σ	0.184	3.85	0.99	0.055
C1-C6	σ	1.518	C5	*σ	0.287	2.16	1.68	0.054
			C1-C2	*σ	0.381	2.86	1.22	0.053
			C2-N15	*σ	0.517	3.37	1.19	0.057
			C5-N10	*σ	0.083	2.58	1.23	0.050
			C2-N15	*σ	0.294	30.32	0.22	0.081
C2-C3	σ	1.543	C1-C2	*σ	0.481	2.69	1.20	0.051
			C3-C4	*σ	0.019	3.64	1.28	0.061
			C4-S7	*σ	0.258	4.08	0.82	0.052
			N15-C18	*σ	0.314	3.70	0.99	0.055
C2-N15	σ	1.566	C1-C6	*σ	0.351	5.27	0.37	0.041
			C3-C4	*σ	0.245	5.66	0.34	0.042
C3-C4	σ	1.466	C2-C3	*σ	0.248	3.18	1.26	0.056
			C2-N15	*σ	0.047	3.06	1.21	0.055
			C4-C5	*σ	0.319	4.33	1.24	0.066
			C5-N10	*σ	0.351	3.24	1.25	0.057
	π	1.571	C2-N15	*π	0.297	29.28	0.24	0.083
C4-C5	σ	1.670	C3-C4	*σ	0.247	4.41	1.30	0.068
			C5-N15	*σ	0.358	2.31	1.22	0.047
C4-S7	σ	1.635	C2-C3	*σ	0.471	3.80	1.16	0.059
C8-C11	π	1.666	C9-N10	*π	0.482	16.62	0.27	0.063
			C12-N16	*π	0.218	29.28	0.24	0.083
C9-N10	π	1.681	C8-C11	*π	0.319	17.76	0.30	0.066
			C13-C14	*π	0.189	16.42	0.33	0.069
C13-C14	π	1.548	C9-N10	*π	0.261	16.9	0.25	0.062
			C12-N16	*π	0.318	30.32	0.22	0.081
LP(1) C5	n	1.912	C1-C6	*π	0.352	47.94	0.16	0.100
			C3-C4	*π	0.469	67.66	0.13	0.102
			C9-N10	*π	0.358	90.03	0.12	0.108
LP(2) S7	n	1.814	C3-C4	*π	0.453	19.28	0.26	0.064
			C8-C11	*π	0.452	19.28	0.26	0.064
LP(1) N10	n	1.871	C4-C5	*σ	0.317	12.26	0.82	0.090
			C8-C9	*σ	0.318	12.26	0.82	0.090

#### Experimental and computational FT-IR interpretation

**Fig. 17:**
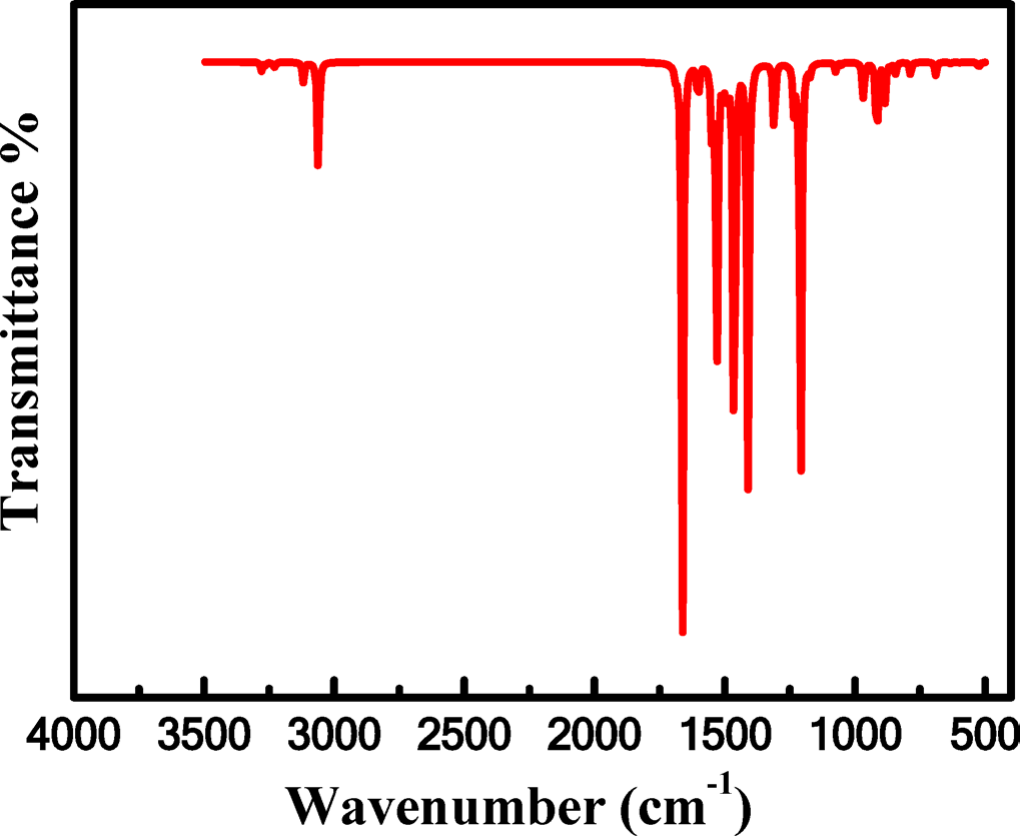
Theoretical FT-IR spectrum of MB dye.

**Fig. 18:**
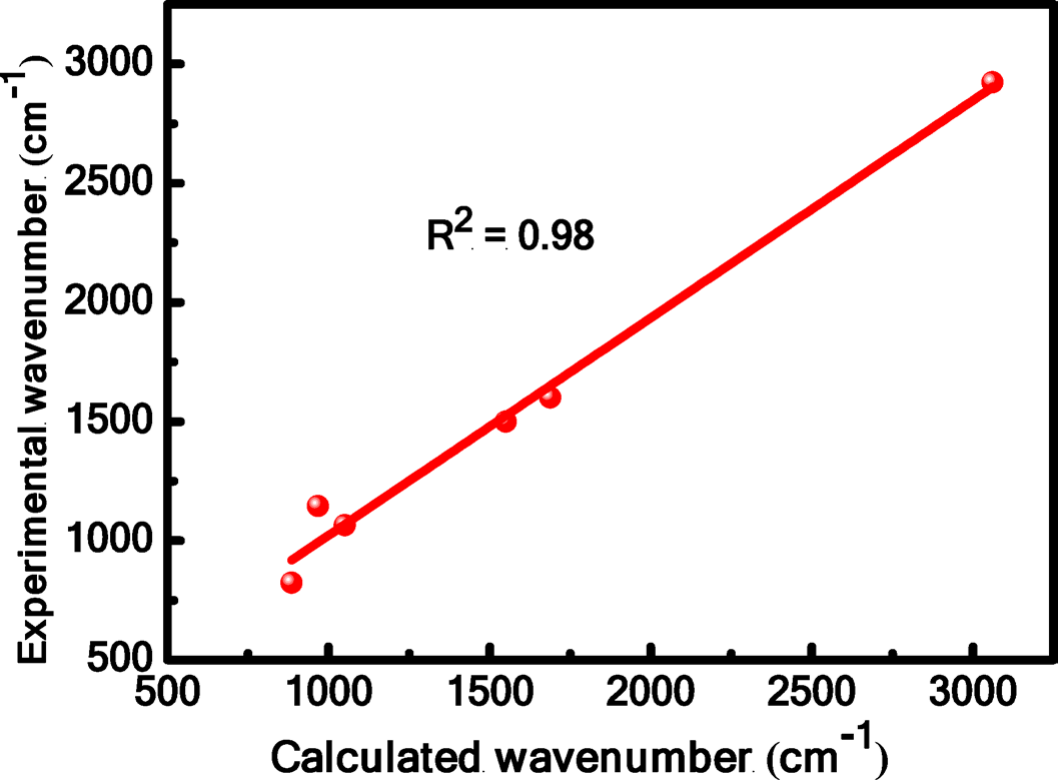
The correlation between the experimental and computed wavenumbers for MB.

**Fig. 19:**
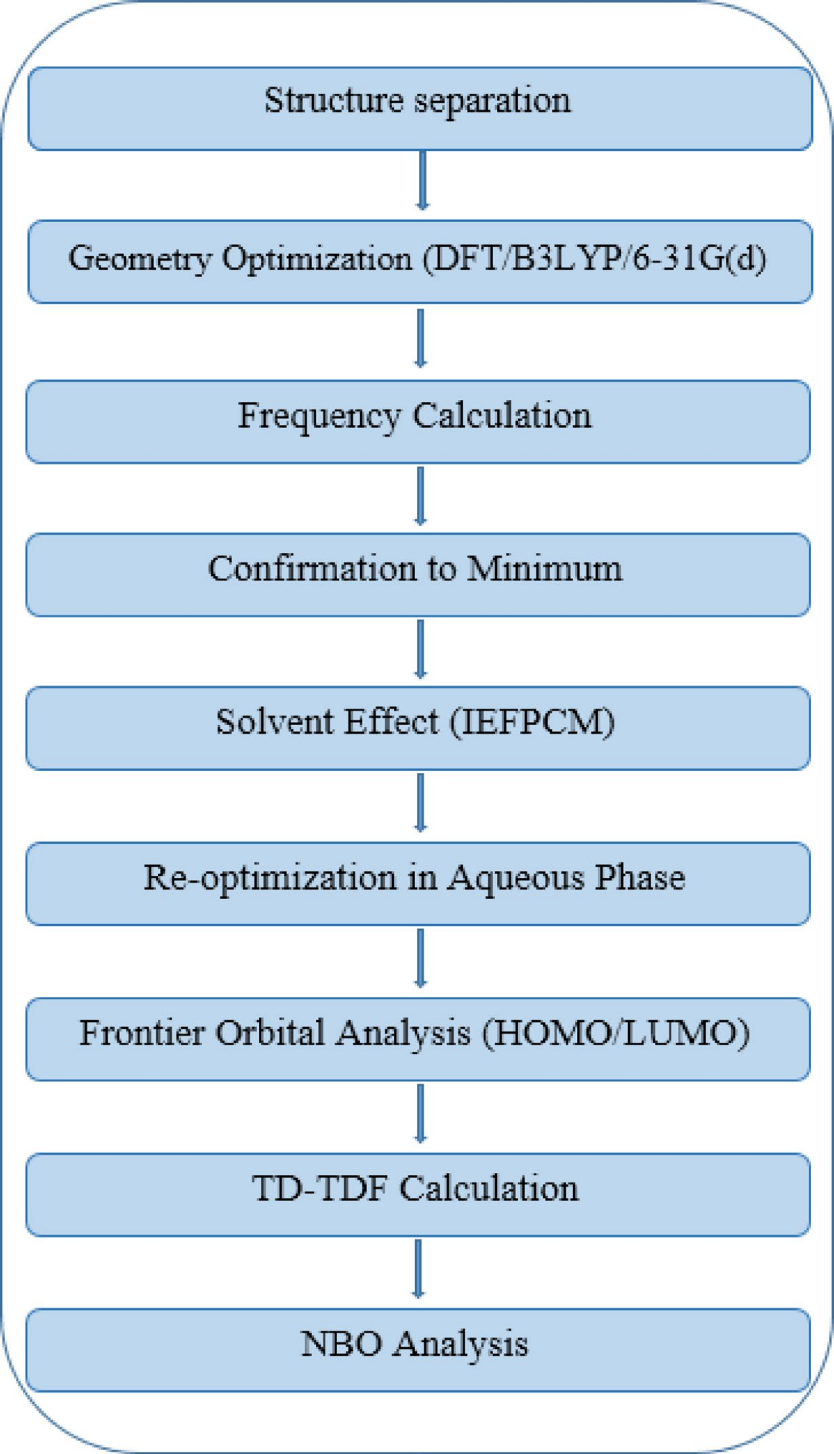
Workflow diagram of all computational steps.

Infrared spectra are a useful tool for detecting functional groups in organic compounds because different functional groups absorb light at different frequencies when the molecules are exposed to light in the infrared region. Because each functional group in the IR spectrum has a distinct fingerprint, we can identify functional groups in organic molecules and compare the frequencies for new compounds to conventional values. This study examined the MB by both the experimental and computational methods, Fig. 17. The 6-31G ++ (d, p) and the B3LYP functional basis sets were used to study the MB spectrum. We found through value comparison that the computational and experimental values are comparable. The calculated asymmetric C-H stretching band appeared as a broad band at 3061 cm^− 1^, which agrees with the experimental value at 2980 cm^− 1^. The C-N band is located at 1146 cm^− 1^, whereas the calculated vibration band is at 1080 cm^− 1^. The calculated stretching vibration of C = C in aromatic rings appearing at 1550 cm^− 1^ is in good agreement with the experimental value at 1500 cm^− 1^. Experimentally, the C = N stretching band of the aromatic ring assigned at 1600 cm^− 1^ fitted well with a calculated value of 1688 cm^− 1^. The C-H out-of-plane stretching band of the aromatic ring is visible at 885 cm^− 1^, while the experimental is seen at 824 cm^− 1^. Additionally, the calculated vibration band occurs at 1051 cm^− 1^, while the experimental C-S-C band of the thiazine ring emerges at 1064 cm^− 1^ ^73^. Using the correlation graph, the relationship between the estimated and experimental wave numbers was evaluated; the value of the linear correlation coefficient (R^2^) was 0.98. There seems to be a tight relationship between the computed results and the experimental data. Excellent agreement between the calculated and observed wavenumbers of MB is demonstrated in Fig. 18. The workflow diagram of all computational steps from structure preparation to electronic and reactivity analysis is provided in Fig. 19, and the main findings are summarized in Table 8.

**Table 8:** The main findings of all computational steps for catalytic reduction of MB.

Parameter/Analysis	Key findings
Geometry optimization	MB structure planarized, optimized in both gas and aqueous phases, water stabilizes planar conformation
HOMO/LUMO energies	HOMO: electron donor, LUMO: electron acceptor, aqueous phase stabilizes orbitals and narrows gap
TD-DFT UV–Vis	Strong π → π* transition at 530.8 nm (HOMO → LUMO)
NBO analysis	Lone pair → π* and π → π* hyperconjugation stabilize aromatic rings; nitrogen and sulfur atoms act as electron donors
Charge distribution	Solvent increases electron delocalization; enhances dipole moment and charge transfer potential
Reaction mechanism insights	HOMO(LBH₄⁻) → LUMO(MB) electron transfer favored; enhanced by higher NaBH₄; pH and temperature modulate adsorption and electron-transfer efficiency

## Conclusion

The CuFeMoO_4_/CMC catalyst is a novel and highly effective catalyst prepared by a facile and cost-effective method by crosslinking CuFeMoO_4_ with CMC polymer using AlCl_3_ as a crosslinker. It has a surface area of 34.88 m^2^g^− 1^. Its catalytic activity was greater than that of the individual components, CuFeMoO_4_ and CMC. The optimum catalytic conditions for the MB reduction were [MB]_o_ = 7.03 mgL^− 1^, [NaBH_4_]_o_ = 41.6 mgL^− 1^, pH = 6, and T = 30 °C. The catalyst exhibited high removal efficiency at pH 6. The incorporation of NaCl in the reaction medium inhibited the reduction rate of MB. The catalyst was reusable effectively for five cycles, with a removal efficiency of 99%, which dropped to 56% at the eighth cycle. Gaussian09 and the 6-31G++(d, p) basis set were employed for the computational analysis. The geometrical structure of MB, quantum chemical parameters, and the effect of the solvent were studied using DFT. The calculations confirmed that MB with a positive charge on the S-atom is more active for the reduction relative to that of a positive charge on the N-atom. Therefore, the S^+^-moiety in the MB molecule is the most advantageous electrophilic site for the reduction of MB by the CuFeMoO_4_/CMC catalyst. The local reactivity indices like hardness, local softness, NBO, TD-DFT, and FT-IR were calculated. The theoretical data match smoothly with the experimental findings. Because the CuFeMoO₄/CMC demonstrated a highly active, stable, and recyclable catalyst for the NaBH₄-driven reduction of methylene blue, it can be regarded as a promising material for real-world wastewater treatment applications that rely on dye reduction.

## Data Availability

All data and materials are available in this article.
